# Advanced whole transcriptome sequencing and artificial intelligence/machine learning (AI/ML) in imiquimod-induced psoriasis-like inflammation of human keratinocytes

**DOI:** 10.37796/2211-8039.1468

**Published:** 2024-12-01

**Authors:** Lii-Tzu Wu, Shih-Chang Tsai, Tsung-Jung Ho, Hao-Ping Chen, Yu-Jen Chiu, Yan-Ru Peng, Ting-Yuan Liu, Yu-Ning Juan, Jai-Sing Yang, Fuu-Jen Tsai

**Affiliations:** a Department of Microbiology, School of Medicine, China Medical University, Taichung, Taiwan; b Department of Biological Science and Technology, China Medical University, Taichung, Taiwan; c Integration Center of Traditional Chinese and Modern Medicine, Hualien Tzu Chi Hospital, Buddhist Tzu Chi Medical Foundation, Hualien, Taiwan; d School of Post-Baccalaureate Chinese Medicine, Tzu Chi University, Hualien, Taiwan; e Department of Biochemistry, Tzu Chi University, Hualien, Taiwan; f Division of Plastic and Reconstructive Surgery, Department of Surgery, Taipei Veterans General Hospital, Taipei, Taiwan; g Department of Surgery, School of Medicine, National Yang Ming Chiao Tung University, Taipei, Taiwan; h Department of Ophthalmology, Taipei City Hospital, Taipei, Taiwan; i Million-Person Precision Medicine Initiative, Department of Medical Research, China Medical University Hospital, Taichung, Taiwan; j Department of Medical Research, China Medical University Hospital, China Medical University, Taichung, Taiwan; k China Medical University Children’s Hospital, Taichung, Taiwan; l Department of Medical Genetics, China Medical University Hospital, Taichung, Taiwan

**Keywords:** Whole transcriptome sequencing, Artificial intelligence/machine learning (AI/ML), Imiquimod (IMQ), Psoriasis, Human keratinocytes

## Abstract

**Introduction:**

Although the HaCaT keratinocyte model has been used in previous research to study the effects of antipsoriatic agents, there is still a lack of comprehensive understanding of the mechanism of imiquimod (IMQ)-induced proliferation and signal transduction in psoriasis-like keratinocytes.

**Objectives:**

This study aimed to investigate the molecular mechanisms and pathways associated with psoriasis-like inflammation caused by IMQ in human keratinocytes.

**Materials and methods:**

HaCaT cells were exposed to different concentrations of IMQ to induce inflammation similar to that observed in psoriasis. Cell viability was evaluated using the MTT assay and cell morphology was examined using phase-contrast microscopy. Gene expression profiles were analyzed through whole transcriptome sequencing, followed by bio-informatics network analysis using IPA software. The GSEA was conducted with the aim of identifying enriched pathways. The expression of key cytokines IL-6 and TNF-α was confirmed by QPCR. Artificial intelligence/machine learning (AI/ML) algorithms were used to predict potential diseases and phenotypes associated with the observed gene profiles.

**Results:**

IMQ treatment demonstrated a substantial positive impact on cell survival without any detectable alterations in the morphology of HaCaT cells. A comprehensive analysis of the entire set of transcribed genes identified 513 genes that exhibited differential expression. Bioinformatics analysis revealed key pathways associated with immune response, cellular proliferation, and cytokine signaling. GSEA identified significant enrichment in the IFN-γ response and JAK-STAT signaling pathways. QPCR analysis confirmed the increased mRNA expression levels of IL-6 and TNF-α in cells treated with IMQ. AI/ML algorithms have identified potential correlations with diseases, such as multiple sclerosis, lympho-proliferative malignancy, and autoimmune disorders.

**Conclusion:**

Our results highlight the importance of specific genes and pathways, particularly those associated with IFN-γ pathway and IL-6/JAK-STAT signaling. AI/ML predictions indicate potential associations with various diseases and provide valuable insights for the development of novel therapeutic approaches for psoriasis and related disorders.

## 1. Introduction

Psoriasis is a chronic autoimmune disease that is not contagious and often coexists with other medical conditions [1]. It is characterized by longlasting symptoms and a tendency to recur, leading to sleep disturbances and concentration difficulties [2]. The clinical manifestations of psoriasis can range from small patches to widespread involvement of the entire body, and can even trigger suicidal thoughts in individuals with mental health issues [3]. Psoriasis is also associated with an increased risk of developing other serious illnesses such as arthritis, metabolic syndrome, cardiovascular disease, crohn’s disease, and respiratory complications [4–6]. The etiology of psoriasis is believed to be multifactorial, involving genetic factors, skin injury, climate, immunodeficiency, certain infections, and the use of specific medications [7–9]. The development of psoriasis is a complex process, and evidence suggests that both innate and adaptive immune systems play a role in the disease [10,11]. Various genetic and environmental factors stimulate immune cells to release inflammatory cytokines, such as TNF-α, IFN-γ, IL-6, IL-12, and IL-23, which in turn cause abnormal growth and development of skin cells called keratinocytes [11–15]. Keratinocytes are activated by Th1 and Th17 cells, resulting in the production of antimicrobial peptides, inflammatory cytokines, and chemokines, which contribute to the characteristic inflammation observed in the skin of patients [16–18]. The involvement of Th17 and Tγδ cells that produce IL-17A is highlighted by variations in genes encoding the IL-23 receptor and IL-12, which have been shown to be crucial for psoriasis development. This disease is characterized by the activation of the IL-12/IL-23/IL-17 cytokine axis [11,17,19–21].

Imiquimod (IMQ; Aldara®) is an immune modulator that is effective in limiting viral infections and inhibiting tumor growth [22–25]. It exerts its primary mode of action through Toll-like receptors (TLR), specifically TLR-7 and TLR-8, which activate NF-κB [26]. NF-κB activation induces cell death in tumor cells and disrupts adenosine receptor signaling pathways [27–32]. Researchers have developed an IMQ-induced mouse model to enhance our understanding of psoriasis, which is widely used in basic research [33–37]. However, the relationship between IMQ-induced psoriasis in mice and human psoriasis in terms of genetics and clinical characteristics is not fully understood [38]. Although previous studies have used the HaCaT keratinocyte model to investigate the effects of antipsoriatic agents, there is still limited knowledge about the mechanism of action of IMQ-induced inflammation, proliferation, differentiation, and signal transduction in psoriasis-like keratinocytes [39–41].

In our previous research, we conducted a genetic analysis using genome-wide association studies (GWAS) to investigate the association between single nucleotide polymorphisms (SNPs), human leukocyte antigen (HLA) genotypes, and psoriasis. The results of our study showed that 153 genes significantly correlated with psoriasis phenotypes. In the Taiwanese population with psoriasis, we found a significant link between HLA-A 02:07 and HLA-C 06:02. The development and progression of psoriasis are influenced by pro-inflammatory cytokines such as IL-12, IL-23, and TNF [42]. To further investigate psoriasis-related mechanisms, in this study we established an *in vitro* model that mimics psoriasis-like proliferation and inflammation induced by IMQ. Subsequently, we performed a gene-level analysis and examined the signaling pathways associated with psoriasis.

## 2. Methods

### 2.1. Chemicals and reagents

The reagents used in this study were obtained from Thermo Fisher Scientific (Waltham, MA, USA): Dulbecco’s Modified Eagle medium (DMEM), fetal bovine serum (FBS), trypsin-EDTA, L-glutamine, penicillin G, and streptomycin. The compounds 3-(4,5-dimethylthiazol-2-yl)-2,5-diphenyltetrazolium bromide (MTT), dimethyl sulfoxide (DMSO), and imiquimod (IMQ) were acquired from Sigma–Aldrich (Merck KGaA, Darmstadt, Germany). Fetal bovine serum (FBS) was obtained from Invitrogen (Carlsbad, CA, USA).

### 2.2. Cell culture

The HaCaT cell line, derived from human normal keratinocytes, was obtained from Elabscience Biotechnology Co., Ltd (Elabscience®; Catalog No.: CL-0090). The cells were cultured in DMEM supplemented with 10% heat-inactivated FBS, penicillin (100 U/ml), and streptomycin (100 μg/mL) and maintained at 37 °C in a 5%CO_2_ atmosphere [43,44].

### 2.3. Cell viability assay

HaCaT cells were cultivated in 96-well plates at a density of 1 × 10^4^ cells/100 μL. They were then exposed to different concentrations of IMQ for 24 h. The concentrations used were 0, 25, 50, and 100 μM. After a 20-h incubation period, a solution of MTT (0.5 mg/mL) was added to each well and incubated at 37 °C for 4 h. The growth media was then extracted, and the formazan crystals were dissolved using 100 μL of DMSO. Absorbance was measured using the SpectraMax iD3 multimode microplate reader (Molecular Devices Ltd., San Jose, CA, USA) at a wavelength of 570/620 nm. Cell viability was calculated as a percentage relative to the control group [45,46].

### 2.4. Cell morphology

HaCaT cells were cultured in 24-well plates at a concentration of 2.5 × 10^5^ cells/mL. They were then exposed to various concentrations (0, 25, 50, and 100 μM) of IMQ for 24 h. The cell images were analyzed using a phase-contrast microscope with a 200× magnification (Leica Microsystems GmbH, Wetzlar, Germany) [44,47].

### 2.5. Whole transcriptome sequencing

Total RNA was isolated using TRIzol ® Reagent (Invitrogen, USA) following the manufacturer’s instructions provided in the manual. RNA was measured using an ND-1000 spectrophotometer (Nanodrop Technology, USA) at a wavelength of 260 nm and assessed for quality using a Bioanalyzer 2100 (Agilent Technology, USA) with an RNA 6000 LabChip kit (Agilent Technology, USA). Library preparation and sequencing processes followed the standard protocol provided by Illumina (Illumina, USA). Library building was performed using the SureSelect XT HS2 mRNA Library Preparation kit from Agilent, USA, and library size selection was carried out using AMPure XP beads from Beckman Coulter, USA. Transcriptome sequencing was performed using the Illumina NovaSeq 6000 system, which utilizes the sequencing-by-synthesis (SBS) method. Welgene Biotech’s process, which utilizes Illumina’s basecalling application bcl2fastq (v2.20), was used to create sequencing data in the form of FASTQ reads. To assess the accuracy of the sequence base obtained fromthe sequencingplatforms,we used the Phred quality score (Q score). We employed the bcl2fastq conversion software v2.20, the official basecalling application developed by Illumina, to convert BCL files from all Illumina sequencing platforms into FASTQ reads. For adaptor cutting and sequence quality trimming, we utilized Trimmomatic (v0.36) with a sliding windowtechnique. Next,we aligned the next-generation sequencing reads to the genomes using HISAT2 software version 2.0.1. Ultimately, the pipeline is applied to the human reference genome (GRCh38.p14) obtained from the UCSC Genome Browser, which may be accessed at the following URL: http://asia.ensembl.org/Homo_sapiens/Info/Index?db=core. Differential expression was studied using StringTie (version 2.1.4) and DEseq (version 1.39.0) with genome bias detection and correction. Genes exhibiting a lowlevel of expression (less than0.3 TPMvalue) in either the treatment or control samples, or in both, were eliminated. Genes that exhibited a p-value of less than 0.05 and a fold change greater than 2.0, were deemed to be significantly differentially expressed. Each stage underwent rigorous quality control and meticulous monitoring [48–50].

### 2.6. Bio-informatics network analysis

The outcome of the volcano plotwas generatedusing the EnhancedVolcano package,which can be accessed at the following URL: https://bioconductor.org/packages/release/bioc/html/EnhancedVolcano.html. The gene set enrichment analysis (GSEA) was executed using the clusterProfiler package. The package can be found at https://www.bioconductor.org/packages/release/bioc/html/clusterProfiler.html. The Gene Set Enrichment Analysis (GSEA) analysis displayed p-values of categories that were less than 0.5. To investigate a molecular network, the Ingenuity Pathway Analysis (IPA) program (version: 107193442; Qiagen Sciences, Inc.) was utilized. The program received data collection consisting of gene symbols and Log2 ratios (IMQ/control). Each gene was associated with a relevant entity in the IPA Knowledge database. Networks and bio-informatics pathways were created using the IPA algorithm [42,51].

### 2.7. Quantitative real time-PCR (QPCR) analysis

HaCaT cells were cultivated in a 75-T flask at a density of 2 × 10^6^ cells/mL. They were then exposed to 0 and 100 μM IMQ, commercially known as Aldara, for 18 h at 37 °C. The EZ2 RNA/miRNA Tissue/Cells Kit (QIAGEN) was used to extract the total RNA. The quantity of RNA samples was assessed using NanoDrop One, a device manufactured by Thermo Fisher Scientific. The RNA samples were reverse-transcribed for 120 min at 37 °C using the High Capacity cDNA Reverse Transcription Kit, following the standard protocol provided by the manufacturer (Applied Biosystems). Quantitative PCR was conducted under the following conditions: a 10-min incubation at 95 °C, followed by 40 cycles of a 15-s incubation at 95 °C and a 1-min incubation at 60 °C. The reaction mixture consisted of 2X Power SYBR Green PCR Master Mix (Applied Biosystems) and 200 nM forward and reverse primers. The assay was performed three times on an Applied Biosystems 7900 Real-Time PCR system, and the fold changes in expression were determined using the comparative CT method. Human glyceraldehyde 3-phosphate dehydrogenase (GAPDH) was used as an endogenous control. The primers used for TNF-α, IL-6, and GAPDH are listed in Table 1 [52].

### 2.8. Artificial intelligence/machine learning (AI/ML) disease pathways prediction analysis

The QIAGEN artificial intelligence/machine learning (AI/ML) database of IPA software (version 107193442; Qiagen Sciences, Inc.) utilizes artificial intelligence/machine learning (AI/ML) disease pathway prediction analysis. It generates over 1500 diseases, phenotypes, and functional pathways. These pathways display primary genes or molecules that influence a specific disease and its related phenotypes. IPA software was used to forecast potential diseases and phenotypes based on genes acquired by RNA sequencing [53,54].

### 2.9. Statistical analysis

Data are presented as mean ± standard deviation. To analyze the variations between groups and conduct multiple comparisons, a one-way analysis of variance was performed, followed by Dunnett’s test. The study utilized SPSS software version 26.0, which was developed in Chicago, IL, USA. The statistical significance level was set at P < 0.001.

## 3. Results

### 3.1. Effect of IMQ on cell viability and morphology of HaCaT cells

**Fig. 1:**
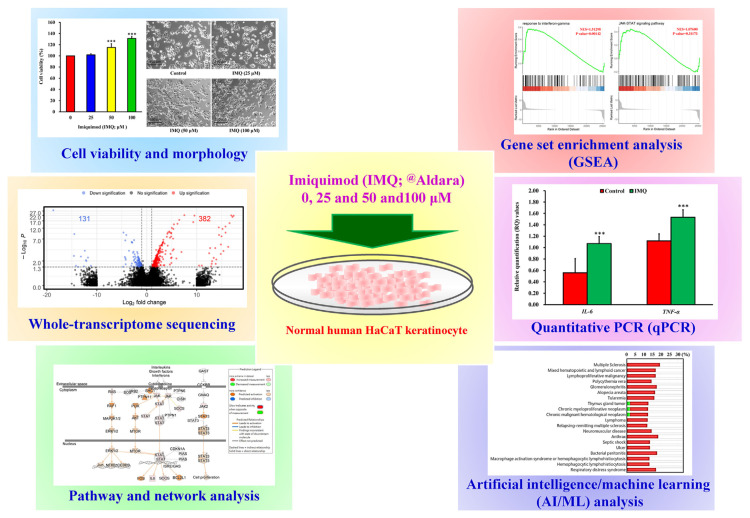
Schematic representation of the study design, assessing the effects of different concentrations of IMQ on cell survival and physical structure. HaCaT keratinocytes were exposed to various concentrations (0, 25, 50, and 100 μM) of IMQ. Cell viability was quantified and images were taken to evaluate changes in morphology. This investigation employed multiple approaches, including whole-transcriptome sequencing, pathway and network analysis, gene set enrichment analysis (GSEA), quantitative PCR (qPCR), and artificial intelligence (AI)/machine learning (ML) analysis.

**Fig. 2:**
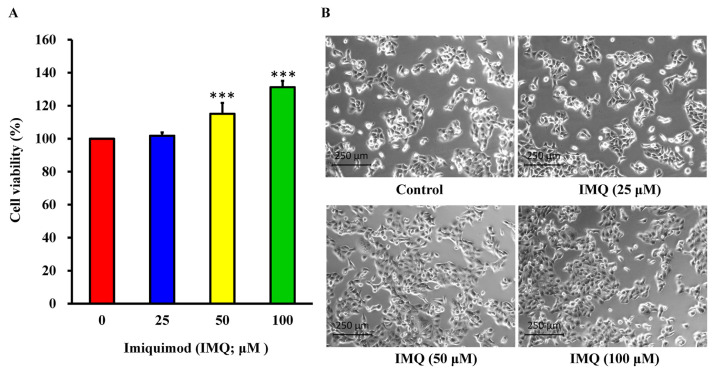
Quantitative assessment of cell viability following imiquimod treatment. (A) The bar graph shows the percentage of viable cells at different concentrations of IMQ (0, 25, 50, and 100 μM). Asterisks (***p < 0.001) indicate statistical significance. (B) Images show the shape and structure of cells under normal conditions and after exposure to IMQ at concentrations of 25, 50, and 100 μM.

The study design is illustrated in Fig. 1. To determine the effect of IMQ on the survival of HaCaT keratinocytes, cell viability was evaluated using the MTT assay. The data in Fig. 2A shows that the application of IMQ at concentrations of 25, 50, and 100 μM resulted in cell viability rates of 101.78 ± 2.04%, 115.05 ± 6.60%, and 131.17 ± 3.78% respectively in HaCaT cells. As demonstrated in Fig. 2B, treatment with IMQ at concentrations of 25, 50, and 100 μM did not alter cell shape. Our finding indicated that IMQ increased cell viability in a concentration-dependent manner.

### 3.2. Whole transcriptome sequencing and bioinformatics network analysis

A comprehensive analysis was performed on the transcriptome sequencing profile to investigate the mechanism of action (MOA) and network of IMQ in HaCaT keratinocytes. The highest dose of IMQ (100 μM) was used for whole transcriptome sequencing. The rawdata fromwhole transcriptome sequencing of HaCaT cells treated with IMQ are provided in Supplementary Table S1 (https://www.biomedicinej.com/cgi/editor.cgi?article=1468&window=additional_files&context=biomedicinej).

**Fig. 3:**
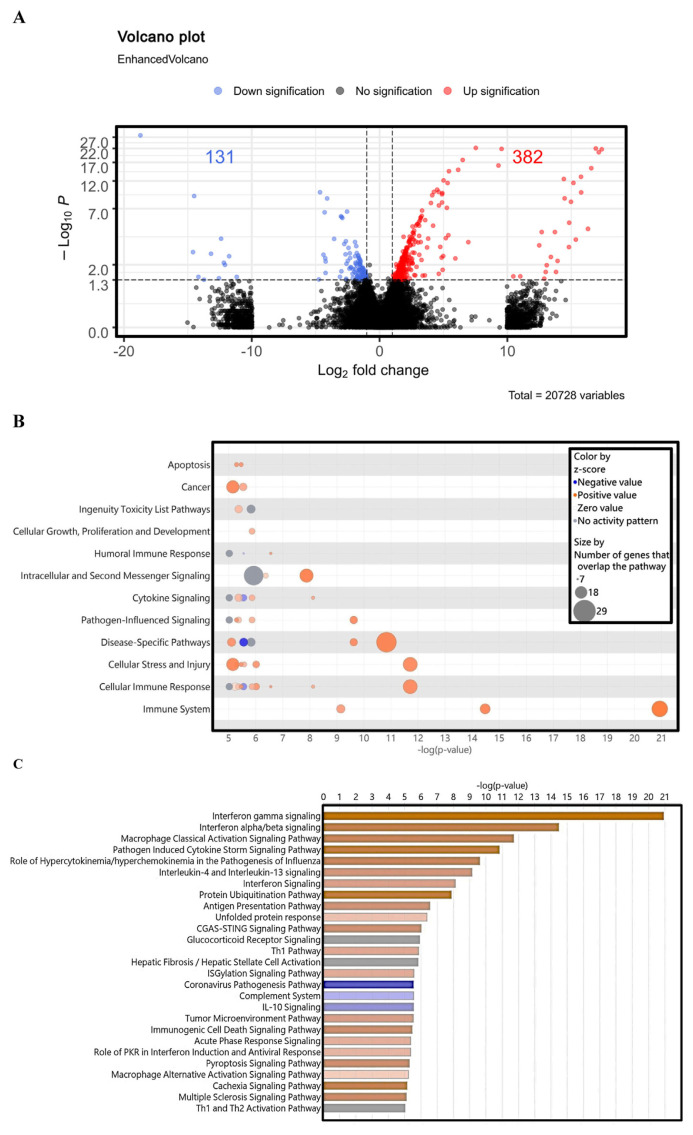
Differences in gene expression after IMQ treatment of HaCaT cells using whole-transcriptome sequencing. (A) Volcano plot is used to display the relationship between the fold change and statistical significance for the identified genes. The y-axis represents increasing statistical significance, whereas the x-axis shows the differential gene expression. The plot was created using the Log2 fold-change values obtained by normalizing IMQ-treated cells with the control treatment. Data points colored red, green, and black correspond to up-regulated genes, down-regulated genes, and genes that did not show a significant difference in expression, respectively. (B) Gene numbers with pathways analysis of full transcriptome sequencing using bio-informatic tools. (C) Graphical representation of the top 25 signaling pathways in HaCaT cells treated with IMQ compared with the control. The number of genes in IMQ-treated HaCaT cells was predicted by cross-analyzing them with pathways using IPA. This prediction was based on the differential gene expression observed in these cells compared with that in the control cells.

Fig. 3A displays volcano plots showing 513 differentially expressed genes. Among these genes, 382 were up-regulated (indicated in red) and 131 were down-regulated (indicated in blue) in IMQ-treated HaCaT keratinocytes. Gene numbers were analyzed along with pathways (Fig. 3B), leading to the prioritization of processes, such as the immune system, cellular immune response, cellular stress and injury, disease-specific pathways, pathogen-influenced signaling, cytokine signaling, intracellular and second messenger signaling, humoral immune response, proliferation and development, cellular growth, ingenuity toxicity list pathways, cancer, and apoptosis.

Fig. 3C provides a graphical overview of the signaling pathways in IMQ-treated HaCaT cells compared with the control. The 25 most important signaling pathways included IFN-γ signaling, interferon alpha/beta signaling, macrophage classical activation signaling pathway, pathogen-induced cytokine storm signaling pathway, role of hypercytokinemia/hyperchemokinemia in the pathogenesis of influenza, IL-4 and IL-13 signaling, interferon signaling, protein ubiquitination pathway, antigen presentation pathway, and unfolded protein response. Other pathways included the CGAS-STING signaling pathway, glucocorticoid receptor signaling, Th1 pathway, hepatic fibrosis, glycosylation signaling pathway, coronavirus pathogenesis pathway, complement system and IL-10 signaling, tumor microenvironment pathway, and immunogenic cell death signaling pathway. The pathways involved also include the signaling of the acute phase response, function of PKR in the activation of interferon production, initiation of antiviral defense mechanisms, pyroptosis signaling pathway, macrophage alternative activation signaling pathway, cachexia signaling pathway, multiple sclerosis signaling pathway, and Th1 and Th2 activation pathways.

**Fig. 4:**
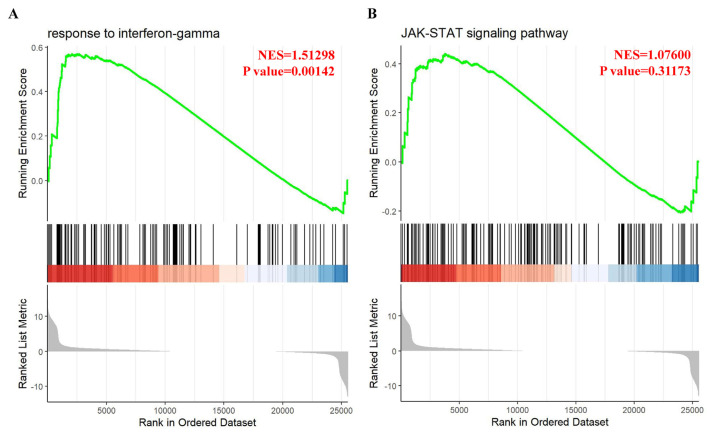
Gene Set Enrichment Analysis (GSEA) for the subset of the most enriched gene sets in imiquimod (IMQ) treatment compared to the control for response to interferon-gamma (IFN-γ) (A) and JAK-STAT signaling pathways (B).

**Fig. 5:**
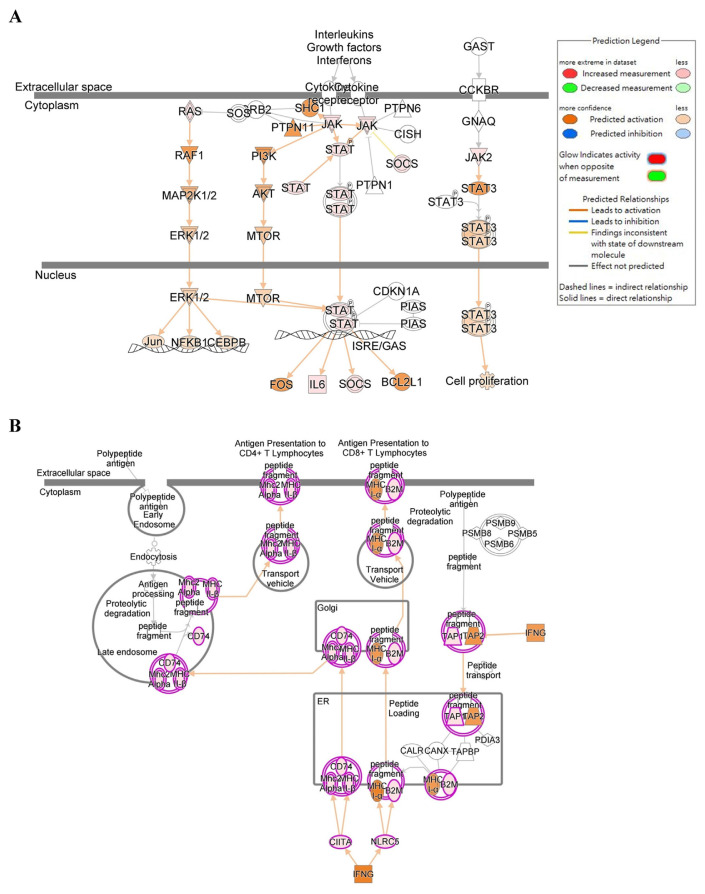
Genes related to the IL-6/JAK/STAT signaling pathway signaling pathway (A) and human leukocyte antigen (HLA) mediated T cell activation pathway (B) were analyzed using the IPA database.

**Fig. 6:**
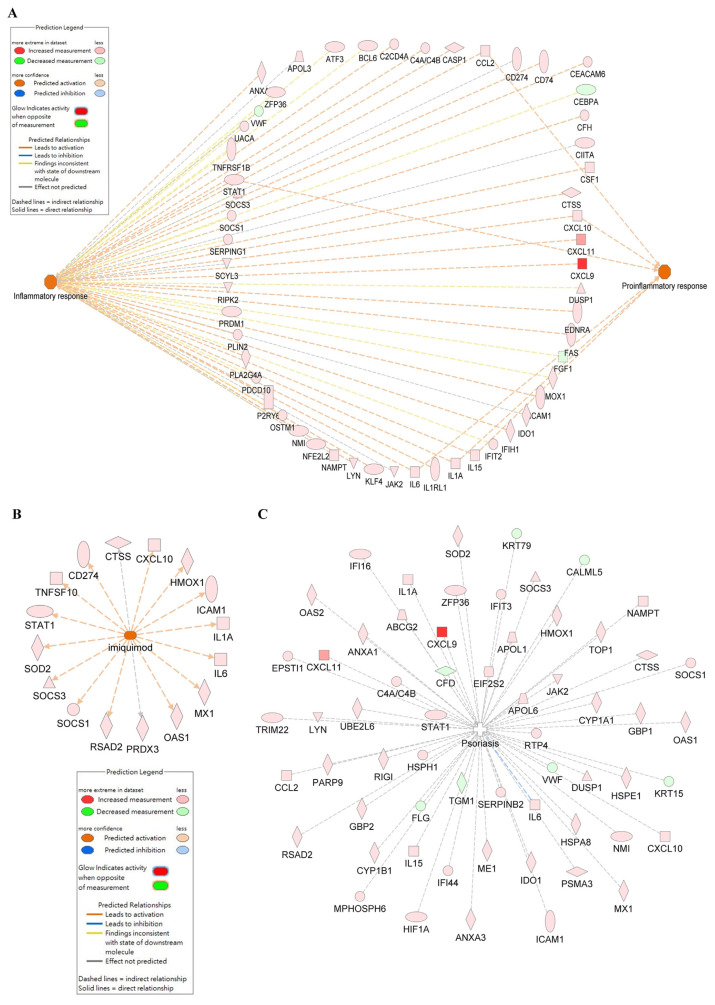
Comprehensive assessment of cellular responses. The genes affected by inflammation and/or pro-inflammatory response (A), imiquimod (IMQ) (B), and psoriasis (C) after IMQ treatment of HaCaT cells was analyzed using IPA. For more detailed information, please refer to the main text.

Gene Set Enrichment Analysis (GSEA) is a bioinformatics analytical method that determines if a pre-defined set of genes exhibits statistically significant and consistent differences between two phenotypes [55]. In this study, GSEA analysis was performed using Gene Ontology categories. The results showed significant enrichment in the response to interferon-gamma category (normalized enrichment score (NES) = 1.51298, P = 0.00142) (Fig. 4A) and the JAK-STAT signaling pathway category (NES = 0.07600, P = 0.31173) (Fig. 4B) in IMQ-treated samples compared to control samples. The genes controlled by the JAK-STAT signaling pathway were analyzed using the IPA database. Our results showed that the IL-6/JAK/STAT signaling pathway (Fig. 5A), and human leukocyte antigen (HLA) mediated T cell activation pathway (Fig. 5B) plays a role in promoting HaCaT keratinocyte proliferation and inflammation by IMQ. Additionally, an analysis was conducted to predict the target genes and their association with inflammation and/or pro-inflammatory responses in HaCaT cells treated with IMQ and psoriasis. The findings are shown in Fig. 6.

### 3.3. Effect of IMQ on IL-6 and TNF-α mRNA levels on HaCaT keratinocytes

**Fig. 7:**
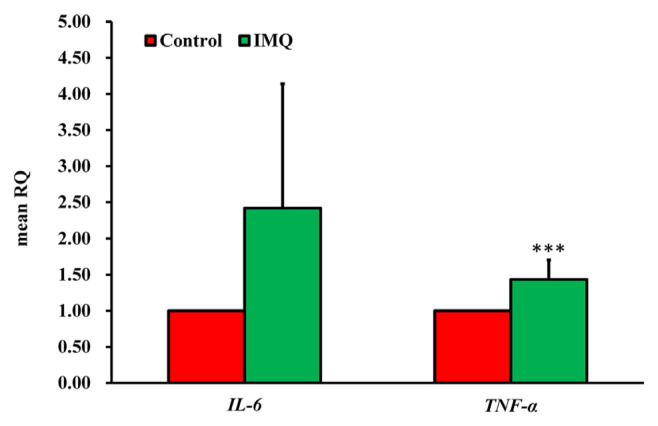
Effect of IMQ on IL-6 and TNF-α mRNA levels in HaCaT keratinocytes analyzed using quantitative real-time PCR (qPCR). The bar graph illustrates the relative quantification (RQ) levels of IL-6 and TNF-α in both the control and IMQ-treated samples. Asterisks (***p < 0.001) indicate statistical significance.

We used quantitative real-time PCR (QPCR) analysis to study how IMQ affects the expression of IL-6 and TNF-α cytokines in HaCaT cells. Fig. 7 shows that when HaCaT cells were treated with 100 μM IMQ, there was an increase in the mRNA expression of IL-6 and TNF-α.

### 3.4. Predict possible diseases and phenotype by artificial intelligence/machine learning (AI/ML)

**Fig. 8:**
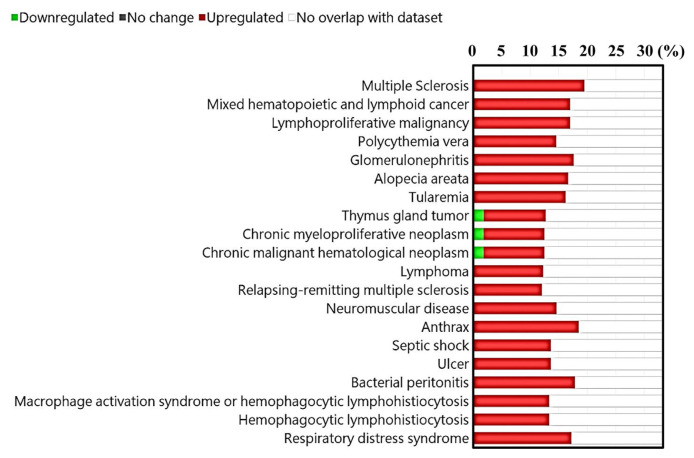
Prediction of possible diseases and phenotypes using artificial intelligence/machine learning (AI/ML) method on IMQ-treated HaCaT keratinocytes. The bar graph illustrates the proportional distribution of treatment effects among various factors.

The results presented in Fig. 8, Table 2, and Supplementary Table S2 (https://www.biomedicinej.com/cgi/editor.cgi?article=1468&window=additional_files&context=biomedicinej) provides a comprehensive understanding of the genetic connections between the important genes in IMQ-treated HaCaT cells and various illnesses by artificial intelligence/machine learning (AI/ML) method. These diseases include multiple sclerosis, mixed hematopoietic and lymphoid cancer, lympho-proliferative malignancy, polycythemia vera, glomerulonephritis, alopecia areata, tularemia, thymus gland tumor, chronic myelo-proliferative neoplasm, chronic malignant hematological neoplasm, lymphoma, relapsing-remitting multiple sclerosis, neuromuscular disease, anthrax, septic shock, ulcer, bacterial peritonitis, macrophage activation syndrome or hemophagocytic lymphohistiocytosis, hemophagocytic lymphohistiocytosis, and respiratory distress syndrome.

## 4. Discussion

Psoriasis is a common skin disease [5] characterized by persistent chronic inflammation and immune-mediated inflammation, which can affect more than just the skin [1,5]. In Taiwan, the occurrence rate of psoriasis is 0.24% [42]. Psoriasis is characterized by the presence of erythematous plaques on silvery scales. These plaques can cause discomfort, irritation, and skin damage [56]. The etiology of psoriasis includes genetic factors [42], environmental factors [57,58], innate and adaptive immune responses [59–61], lifestyle choices [6,62,63], and therapeutic drugs [6,62]. The pathogenesis of psoriasis involves several key processes: (1) excessive proliferation of keratinocytes, fibroblasts, and endothelial cells, (2) activation of the immune system, (3) initiation of an inflammatory response, and (4) stimulation of new blood vessel development (neovascularization or angiogenesis) [5,42,64]. To investigate the causes of psoriasis and identify new therapies, it is important to develop suitable *in vitro* and *in vivo* models of psoriasis [42]. This study aimed to create an *in vitro* model of psoriasis-like inflammation in keratinocytes by using imiquimod.

In this study, the application of IMQ greatly boosted the viability of HaCaT keratinocytes, as demonstrated in Fig. 2A. Fig. 2B shows that IMQ treatment did not result in significant cell death; instead, an increase in the number of cells was observed. This *in vitro* model offers a potential explanation for the atypical growth and inflammation of keratinocytes observed in psoriasis. Our findings are consistent with those of previous research in which HaCaT cells were treated with IMQ (100 μM) to induce inflammation and proliferation resembling psoriasis [40]. IMQhas recently shown potential for the treatment of various dermatological diseases [65,66]. Aldara™, a topical creamcontaining 5% IMQ, has been approved by the FDA as an immune response agent in clinical settings. IMQ is indicated for the treatment of external anogenital warts and basal cell carcinomas [67]. The Mechanism of Action (MOA) of IMQ involves its binding to Toll-like receptors, specifically TLR7/TLR8, in humans. This binding interferes with adenosine receptor (AR) signaling and activation of nuclear factor-kappa B (NF-kB) [68,69]. Activation of protein activity triggers the generation of proinflammatory cytokines and chemokines, leading to an active immune response through the interaction of antigen-presenting cells (APC) with MHC molecules [68,70,71]. Our findings also indicate that certain genes in the NF-kB signaling pathway (CASP1, CD74, EDNRA, GPR89A/GPR89B, HMOX1, IFIT5, IL1A, IRF1, RIPK2, STAT1, TICAM2, TRIM22, TRIM5, UACA, UBD, and ZC3HAV1) as well as MHC molecules (HLA-DMB, HLA-DRA, and HLA-DPA1) are involved in regulating the immune response in HaCaT cells treated with IMQ. The results are summarized in Supplementary Table S1 (https://www.biomedicinej.com/cgi/editor.cgi?article=1468&window=additional_files&context=biomedicinej).

A previous study developed an *in vitro* model of psoriasis using differentiated HaCaT cells generated using IMQ. This model was used to quickly test potential drugs for psoriasis treatment [40]. The cause of psoriasis-like inflammation induced by IMQ was comparable to that observed in a mouse model of psoriasis [30]. To assess the potential signaling pathways of IMQ in HaCaT cells, a comprehensive analysis of the entire transcriptome was conducted in both the IMQ-exposed and control groups. The data presented in Fig. 3 demonstrates a significant correlation between IMQ-induced changes in gene expression and cellular proliferation, anti-apoptotic processes, and inflammatory cytokine production. In our bio-informatics network analysis, we categorized these functions according to distinct gene expression patterns, including: (1) immune responses (immune system, cellular immune response, humoral immune response, and cytokine signaling); (2) inflammatory signaling (signaling influenced by pathogens and cytokine signaling); (3) Cellular proliferation and cellular death (proliferation and expansion of cells, growth and programmed cell death.); and (4) Diseases or other conditions (such as disease-specific pathways, cancer, cellular stress and damage, secondary messenger signaling, and the ingenuity toxicity list pathway). GSEA considers variations in the number of gene sets and correlations between gene sets and the expression dataset. This technique uses a combination of statistical approaches to aggregate data from individual genes into a gene set. This allows the identification of circumstances in which all genes in a pre-determined set exhibit minor but coordinated change [55]. Fig. 4 demonstrates that GSEA analysis revealed a notable increase in enrichment in the response to IFN-γ and JAK-STAT signaling pathways in the IMQ-treatment group compared to the control group. To the best of our knowledge, our study is the first to document that IMQ causes psoriasis-like inflammation in keratinocytes. The results of the associated genes involved in inflammatory reactions, IMQ treatment, and psoriasis are shown in Fig. 6. This study presents evidence that confirms the importance of the IL-6/JAK/STAT signaling pathway and human leukocyte antigen (HLA) mediated T cell activation pathway in various physiological processes and its involvement in psoriatic-like cell formation (Fig. 5).

The etiology of psoriasis involves up-regulation of several pro-inflammatory cytokines, including IL-6, IL-8, IFN-γ, and TNF-α. The correlation between IL-6 and TNF-α, and the development of psoriasis has been widely recognized [72,73]. Figs. 5 and 6 presents the findings that provide evidence for a model explaining keratinocyte inflammation in psoriasis. This model suggests that a particular group of inflammatory products are produced at elevated levels. Moreover, certain inflammatory cytokines, such as IL-6 and TNF-α, were significantly increased owing to mRNA up-regulation. However, our results did not demonstrate an increase in IFN-γ levels, as observed in both whole transcriptome sequencing and QPCR (data not shown). Consequently, we inferred that the increase in IL-6 and TNF-α level was a result of JAK-STAT activation (Fig. 7). This study differs from previous research [40].

Constructing models using artificial intelligence/machine learning (AI/ML) and making predictions about potential diseases and phenotypes is a significant approach in the fields of bio-informatics and artificial intelligence (AI) [74–76]. Polygenic risk scores (PRS) are frequently employed in clinical settings to evaluate an individual’s genetic susceptibility to a specific disease or trait relative to similar groups [77]. In this study, we utilized artificial intelligence/machine learning (AI/ML) to predict potential illnesses and phenotypes in a model of IMQ-induced psoriasis-like inflammation in keratinocytes. The findings presented in Fig. 8, Table 2 and Supplementary Table S2 (https://www.biomedicinej.com/cgi/editor.cgi?article=1468&window=additional_files&context=biomedicinej) indicate that autoimmune diseases such as multiple sclerosis, alopecia areata, and relapsing-remitting multiple sclerosis, as well as cancer types such as mixed hematopoietic and lymphoid malignancy, are significant diseases associated with IMQ-induced psoriasis-like inflammation in keratinocytes. This finding further validates the notion that genes associated with psoriasis may play a role in the development of other autoimmune or cancer-related conditions.

## 5. Conclusion

The current study aimed to provide a more comprehensive understanding of the characterization and mechanism of action (MOA) of keratinocytes treated with IMQ using an *in vitro* model. Additionally, recently discovered genes and their associated network pathways can be used to assess the effectiveness of potential drugs for psoriasis and other skin conditions. This study offers valuable insights into the potential implications of improving dermatological treatment.

## Supplementary Information





## Figures and Tables

**Table 1:** Primers list of quantitative real time-PCR (QPCR) analysis.

Primer Name	Primer sequence
Human IL6-F	GTACATCCTCGACGGCATCTC
Human IL6-R	GTGCCTCTTTGCTGCTTTCAC
Human TNF-α-F	CTCGAACCCCGAGTGACAA
Human TNF-α-R	TTGGCCAGGAGGGCATT
Human GAPDH-F	ACACCCACTCCTCCACCTTT
Human GAPDH-R	TAGCCAAATTCGTTGTCATACC

**Table 2:** Artificial intelligence/machine learning (AI/ML) analysis is used to predict potential disease pathways and phenotypes of IMQ-induced psoriasis-like inflammation in keratinocytes.

My pathways	−Log (p-value)	Ratio	Down-regulated	No change	Up-regulated	No overlap with dataset	Molecules
Multiple Sclerosis	6.38	0.195	0/41 (0%)	0/41 (0%)	8/41 (20%)	33/41 (80%)	FAS, MX1, OAS1, RSAD2, SAMHD1, TAP1, TICAM2, WARS1
Mixed hematopoietic and lymphoid cancer	5.9	0.17	0/47 (0%)	0/47 (0%)	8/47 (17%)	39/47 (83%)	CCL2, CYP1A1, CYP1B1, IFT57, NMI, SERPINB2, TNFSF10, XAF1
Lymphoproliferative malignancy	5.9	0.17	0/47 (0%)	0/47 (0%)	8/47 (17%)	39/47 (83%)	CCL2, CYP1A1, CYP1B1, IFT57, NMI, SERPINB2, TNFSF10, XAF1
Polycythemia vera	4.77	0.146	0/48 (0%)	0/48 (0%)	7/48 (15%)	41/48 (85%)	IRF1, JAK2, NLRC5, STAT1, TNFSF10, UBE2L6, XAF1
Glomerulonephritis	4.65	0.176	0/34 (0%)	0/34 (0%)	6/34 (18%)	28/34 (82%)	HMOX1, IFIH1, IL6, LYN, NFE2L2, SOCS1
Alopecia areata	4.5	0.167	0/36 (0%)	0/36 (0%)	6/36 (17%)	30/36 (83%)	APOL6, BCL6, IL15, IRF1, JAK2, STAT1
Tularemia	4.43	0.162	0/37 (0%)	0/37 (0%)	6/37 (16%)	31/37 (84%)	CASP1, CCL2, IL1A, IL6, IRF1, STAT1
Thymus gland tumor	4.37	0.127	1/55 (2%)	0/55 (0%)	6/55 (11%)	48/55 (87%)	CASP1, HSPE1, IFT57, NMI, SERPINB2, SSTR5, XAF1
Chronic myelo-proliferative neoplasm	4.32	0.125	1/56 (2%)	0/56 (0%)	6/56 (11%)	49/56 (88%)	EIF2S2, EMC2, IFIH1, IRF1, JAK2, NOTCH3, RIGI
Chronic malignant hematological neoplasm	4.32	0.125	1/56 (2%)	0/56 (0%)	6/56 (11%)	49/56 (88%)	EIF2S2, EMC2, IFIH1, IRF1, MLF1, NOTCH3, RIGI
Lymphoma	4.27	0.123	0/57 (0%)	0/57 (0%)	7/57 (12%)	50/57 (88%)	CCL2, IFT57, NBN, NMI, SERPINB2, TNFSF10, XAF1
Relapsing-remitting multiple sclerosis	4.22	0.121	0/58 (0%)	0/58 (0%)	7/58 (12%)	51/58 (88%)	EPSTI1, OAS1, RSAD2, SAMHD1, TAP1, TICAM2, WARS1
Neuromuscular disease	4.17	0.146	0/41 (0%)	0/41 (0%)	6/41 (15%)	35/41 (85%)	CTSS, EPSTI1, OAS1, PDCD1LG2, STAT1, WARS1
Anthrax	4.06	0.185	0/27 (0%)	0/27 (0%)	5/27 (19%)	22/27 (81%)	CASP1, CXCL10, IDO1, NLRC5, STAT1
Septic shock	3.99	0.136	0/44 (0%)	0/44 (0%)	6/44 (14%)	38/44 (86%)	ATF3, ICAM1, IL1A, IRF1, RIPK2, STAT1
Ulcer	3.99	0.136	0/44 (0%)	0/44 (0%)	6/44 (14%)	38/44 (86%)	APOL6, CASP1, CXCL10, PLA2G4A, SOCS1, STAT1
Bacterial peritonitis	3.98	0.179	0/28 (0%)	0/28 (0%)	5/28 (18%)	23/28 (82%)	ATF3, CCL2, IL6, P2RY6, RIPK2
Macrophage activation syndrome or hemophagocytic lymphohistiocytosis	3.94	0.133	0/45 (0%)	0/45 (0%)	6/45 (13%)	39/45 (87%)	CCL2, IL1A, IL6, JAK2, STAT1, STX11
Hemophagocytic lymphohistiocytosis	3.94	0.133	0/45 (0%)	0/45 (0%)	6/45 (13%)	39/45 (87%)	CCL2, IL1A, IL6, JAK2, STAT1, STX11
Respiratory distress syndrome	3.9	0.172	0/29 (0%)	0/29 (0%)	5/29 (17%)	24/29 (83%)	JAK2, NFE2L2, NMI, RIGI, TNFRSF1B
Hemophagocytic syndrome	3.88	0.13	0/46 (0%)	0/46 (0%)	6/46 (13%)	40/46 (87%)	CCL2, IL1A, IL6, JAK2, STAT1, STX11
Small-cell carcinoma	3.78	0.125	0/48 (0%)	0/48 (0%)	6/48 (13%)	42/48 (88%)	B2M, CD274, EIF2S2, MLF1, SOD2, TOP1
Uveitis	3.76	0.161	0/31 (0%)	0/31 (0%)	5/31 (16%)	26/31 (84%)	ANXA1, IRF1, PDCD1LG2, STAT1, TNFRSF1B
Myasthenia gravis	3.76	0.161	0/31 (0%)	0/31 (0%)	5/31 (16%)	26/31 (84%)	CD55, CTSS, IL1A, PDCD1LG2, STAT1
Shock Response	3.73	0.122	0/49 (0%)	0/49 (0%)	6/49 (12%)	43/49 (88%)	ATF3, ICAM1, IRF1, RIPK2, STAT1, TNFRSF1B
Development of urinary tract tumor	3.68	0.12	0/50 (0%)	0/50 (0%)	6/50 (12%)	44/50 (88%)	ATF3, HIF1A, HSPE1, KLF4, NAE1, NFE2L2
Chronic myeloid leukemia	3.63	0.118	1/51 (2%)	0/51 (0%)	5/51 (10%)	45/51 (88%)	EIF2S2, EMC2, IFIH1, NOTCH3, RIGI, SOCS3
Infection by *Mycobacterium bovis*	3.56	0.147	0/34 (0%)	0/34 (0%)	5/34 (15%)	29/34 (85%)	IL15, IL1A, IRF1, PDCD1LG2, STAT1
Mediastinal neoplasm	3.54	0.113	0/53 (0%)	0/53 (0%)	6/53 (11%)	47/53 (89%)	CASP1, HSPE1, IFT57, NMI, SERPINB2, XAF1
Growth of tumor	3.5	0.143	0/35 (0%)	0/35 (0%)	5/35 (14%)	30/35 (86%)	CSF1, HIF1A, IL6, NMI, SLC2A3

## List of Abbreviations

AI: Artificial intelligence
DMEM: Dulbecco’s Modified Eagle medium
DMSO: Dimethyl sulfoxide
FBS: Fetal bovine serum
GSEA: Gene Set Enrichment Analysis
GWAS: Genome-wide association studies
HLA: Human leukocyte antigen
IFN-γ: Interferon-gamma
IL: Interleukin
IMQ: Imiquimod
IPA: Ingenuity Pathway Analysis
ML: Machine Learning
MOA: Mechanism of action
MTT: 3-(4,5-dimethylthiazol-2-yl)-2,5-diphenyltetrazolium bromide
NES: Normalized enrichment score
NF-κB: Nuclear factor-kappa B
PRS: Polygenic risk scores
QPCR: Quantitative real-time PCR
SNPs: Single nucleotide polymorphisms
TNF-α: Tumor Necrosis Factor-α
TLR: Toll-like receptors
