# Supplementary material for: Advanced whole transcriptome sequencing and artificial intelligence/machine learning (AI/ML) in imiquimod-induced psoriasis-like inflammation of human keratinocytes
Source: Biomedicine (Taipei). 2024 Dec 1;14(4):36–50. doi: 10.37796/2211-8039.1468 (PMC11703395; doi:10.37796/2211-8039.1468)
Supplement: Supplementary file 1 [file SupplementaryTableS1.pdf]

| Supplementary Table S1: Raw data of whole transcriptome sequencing on IMQ-treated HaCaT cells. | | | | |
|---|---|---|---|---|
| Gene name | TPM: IMQ | TPM: Control | Log2 ratio (IMQ/Control) | P value |
| TMEM189-UBE2V1 | 17.0955640 | 0.0001000 | 17.3832625 | 2.27E-22 |
| CXCL9 | 14.5614450 | 0.0001000 | 17.1517940 | 3.04E-21 |
| HNRNPUL2-BSCL2 | 12.3706170 | 0.0001000 | 16.9165579 | 1.03E-22 |
| LGALS17A | 9.6831630 | 0.0001000 | 16.5631908 | 5.39E-16 |
| TRMT112P6 | 8.1063140 | 0.0001000 | 16.3067584 | 2.41E-05 |
| CXCL11 | 5.5955920 | 0.0001000 | 15.7720032 | 2.31E-10 |
| APOL4 | 5.5441780 | 0.0001000 | 15.7586860 | 1.03E-13 |
| AC004069.1 | 4.1856150 | 0.0001000 | 15.3531520 | 2.16E-04 |
| AC131160.1 | 3.6758730 | 0.0001000 | 15.1657993 | 2.73E-12 |
| AC093525.2 | 3.2178570 | 0.0001000 | 14.9738126 | 9.71E-09 |
| RPL17-C18orf32 | 2.9294660 | 0.0001000 | 14.8383501 | 5.61E-06 |
| USP30-AS1 | 2.8893490 | 0.0001000 | 14.8184569 | 8.12E-04 |
| BLOC1S5-TXNDC5 | 2.2850840 | 0.0001000 | 14.4799596 | 2.63E-09 |
| AL035461.3 | 2.1882780 | 0.0001000 | 14.4175084 | 3.58E-13 |
| TEX29 | 1.5580730 | 0.0001000 | 13.9274752 | 6.17E-03 |
| C20orf141 | 1.4304970 | 0.0001000 | 13.8042289 | 2.27E-02 |
| AC025165.3 | 1.3478130 | 0.0001000 | 13.7183327 | 4.76E-05 |
| LINC00941 | 1.0836650 | 0.0001000 | 13.4036312 | 3.66E-03 |
| AC091564.7 | 0.8637090 | 0.0001000 | 13.0763296 | 1.05E-02 |
| MFSD2B | 0.8176860 | 0.0001000 | 12.9973312 | 2.27E-02 |
| AD000671.1 | 0.7562130 | 0.0001000 | 12.8845769 | 4.46E-02 |
| MIR34AHG | 0.6590450 | 0.0001000 | 12.6861613 | 4.76E-05 |
| C2CD4A | 0.5768080 | 0.0001000 | 12.4938755 | 6.08E-04 |
| AC138028.2 | 0.2066540 | 0.0001000 | 11.0130016 | 3.56E-02 |
| MROH7-TTC4 | 0.1414890 | 0.0001000 | 10.4664742 | 3.56E-02 |
| GBP5 | 31.8286170 | 0.0425440 | 9.5471531 | 1.62E-22 |
| CXCL10 | 23.5828110 | 0.0373250 | 9.3033777 | 8.98E-17 |
| IDO1 | 139.2691800 | 0.7533940 | 7.5302558 | 6.15E-23 |
| AC118281.1 | 0.5832500 | 0.0047390 | 6.9433879 | 3.47E-04 |
| GBP1 | 322.1656490 | 3.5480770 | 6.5046215 | 1.62E-18 |
| UBD | 110.4806440 | 1.5545960 | 6.1511101 | 1.55E-15 |
| MIR222HG | 1.4313730 | 0.0234940 | 5.9289635 | 4.45E-03 |
| CIITA | 44.9516600 | 1.0456680 | 5.4258776 | 3.82E-15 |
| CAND2 | 0.8792270 | 0.0210430 | 5.3848234 | 9.18E-05 |
| BATF2 | 33.0993840 | 0.8269380 | 5.3228814 | 2.89E-12 |
| SERPING1 | 6.9142890 | 0.1796910 | 5.2659908 | 7.67E-08 |
| TMPRSS3 | 1.9282560 | 0.0531350 | 5.1814903 | 1.65E-04 |
| GBP4 | 96.2121050 | 2.9889090 | 5.0085275 | 7.67E-13 |
| AC010132.3 | 1.8914900 | 0.0599360 | 4.9799565 | 3.20E-03 |
| SNURF | 1.9340630 | 0.0631980 | 4.9356121 | 1.81E-02 |
| CD274 | 12.2393990 | 0.4064970 | 4.9121442 | 3.09E-10 |
| GBP1P1 | 10.8341890 | 0.3652420 | 4.8905947 | 1.22E-08 |
| AL137782.1 | 7.8852530 | 0.2668400 | 4.8851102 | 5.19E-10 |
| AC125257.1 | 0.7598360 | 0.0262600 | 4.8547492 | 3.99E-03 |
| AC015813.2 | 1.4871260 | 0.0552810 | 4.7495994 | 7.87E-04 |
| IL18BP | 24.8658890 | 0.9963350 | 4.6413933 | 3.44E-08 |
| ACKR4 | 0.5851430 | 0.0235940 | 4.6322973 | 2.75E-02 |
| AC023055.1 | 12.2505540 | 0.4984800 | 4.6191676 | 1.89E-10 |
| GBP2 | 79.1949840 | 3.4828720 | 4.5070597 | 6.85E-11 |
| ICAM1 | 191.5926510 | 10.0515970 | 4.2525456 | 1.88E-10 |
| RMRP | 34.1518480 | 1.8749690 | 4.1870250 | 1.16E-05 |
| STAT1 | 539.2734380 | 31.0383530 | 4.1188931 | 7.43E-10 |
| AC242842.3 | 4.6309790 | 0.2854970 | 4.0197697 | 1.48E-08 |
| PDCD1LG2 | 2.7665100 | 0.1981050 | 3.8037299 | 1.81E-04 |
| CCDC144A | 0.3851130 | 0.0292620 | 3.7181815 | 8.68E-04 |
| NLRC5 | 57.9417190 | 4.8664340 | 3.5736656 | 2.89E-06 |
| ANKRD1 | 0.9646590 | 0.0827810 | 3.5426475 | 3.23E-02 |
| ERP27 | 2.2278870 | 0.2155150 | 3.3698159 | 3.31E-02 |
| GBP3 | 81.0038760 | 8.0793880 | 3.3256730 | 1.85E-07 |

| | | | | |
|---|---|---|---|---|
| IRF9 | 71.6560060 | 7.2857510 | 3.2979380 | 9.06E-07 |
| C4B | 2.4147430 | 0.2483220 | 3.2815857 | 5.48E-03 |
| HSD17B2 | 60.7927020 | 6.5204560 | 3.2208534 | 1.51E-06 |
| CFAP298-TCP10L | 1.1524740 | 0.1237130 | 3.2196652 | 3.41E-04 |
| XAF1 | 24.3556420 | 2.6240160 | 3.2144076 | 1.40E-06 |
| AP000944.5 | 19.7914260 | 2.1861190 | 3.1784317 | 2.75E-06 |
| TENT5C | 0.9347190 | 0.1085210 | 3.1065585 | 2.69E-03 |
| PARP9 | 118.5356830 | 13.8219190 | 3.1002916 | 1.16E-05 |
| EDNRA | 3.8963440 | 0.4552460 | 3.0974028 | 1.06E-04 |
| AC022034.1 | 15.4206940 | 1.8097190 | 3.0910301 | 4.34E-06 |
| HLA-DRA | 8.8756950 | 1.0991800 | 3.0134324 | 3.70E-04 |
| WARS1 | 4589.5429690 | 581.4541020 | 2.9806133 | 7.35E-06 |
| ETV7 | 109.3051680 | 14.2106120 | 2.9433210 | 8.24E-06 |
| PAK3 | 0.5717510 | 0.0768920 | 2.8944816 | 1.28E-02 |
| APOL2 | 149.9976500 | 20.8116970 | 2.8494734 | 1.13E-05 |
| AC005520.1 | 4.1758220 | 0.5874880 | 2.8294289 | 3.10E-04 |
| AC000120.4 | 0.9876590 | 0.1421360 | 2.7967410 | 3.24E-02 |
| APOBEC3D | 7.3372960 | 1.1079620 | 2.7273401 | 2.02E-03 |
| ZNF814 | 0.5359220 | 0.0814420 | 2.7181781 | 3.14E-02 |
| APOL3 | 100.7975850 | 15.4557860 | 2.7052421 | 3.54E-05 |
| TNFRSF1B | 7.5741710 | 1.1885620 | 2.6718708 | 2.23E-04 |
| SAMD9L | 13.2739110 | 2.0832290 | 2.6717002 | 7.93E-05 |
| AC007192.1 | 26.3129810 | 4.2054360 | 2.6454474 | 5.59E-05 |
| RN7SK | 53.3677900 | 8.5361090 | 2.6443188 | 5.23E-04 |
| IFIT3 | 213.6847230 | 35.3179210 | 2.5970114 | 5.42E-05 |
| AC048338.2 | 0.9668600 | 0.1600990 | 2.5943427 | 8.58E-03 |
| CTSS | 9.5561520 | 1.5835510 | 2.5932665 | 1.89E-04 |
| IL6 | 10.1850210 | 1.6938800 | 2.5880454 | 1.92E-03 |
| IRF1 | 837.8540650 | 140.2164000 | 2.5790439 | 1.40E-05 |
| TMEM140 | 2.8235400 | 0.4761280 | 2.5680837 | 8.26E-03 |
| MEMO1P1 | 8.6380650 | 1.4786700 | 2.5464081 | 4.78E-03 |
| PLSCR4 | 4.9045580 | 0.8560410 | 2.5183713 | 6.28E-03 |
| TRIM22 | 47.2880550 | 8.3510380 | 2.5014484 | 1.39E-04 |
| TMEFF1 | 2.6336570 | 0.4755100 | 2.4695199 | 7.87E-03 |
| BCL2L14 | 2.0711420 | 0.3744370 | 2.4676316 | 1.82E-02 |
| PSMA4 | 217.5676570 | 40.0523030 | 2.4415070 | 5.63E-04 |
| DUS4L-BCAP29 | 1.9225050 | 0.3572310 | 2.4280582 | 4.08E-03 |
| CMPK2 | 14.6291010 | 2.7793800 | 2.3960061 | 6.31E-04 |
| PARP14 | 81.1504820 | 15.6465640 | 2.3747538 | 2.10E-04 |
| RPPH1 | 9.5201450 | 1.8553600 | 2.3592844 | 4.40E-02 |
| HAPLN3 | 59.3462750 | 11.5793850 | 2.3575988 | 1.22E-04 |
| TRIM69 | 75.3486020 | 14.7488250 | 2.3529807 | 2.98E-04 |
| ANXA10 | 3.6668550 | 0.7202540 | 2.3479655 | 1.64E-02 |
| PMAIP1 | 79.3139040 | 15.6773600 | 2.3388912 | 3.38E-04 |
| GIMAP2 | 5.7084320 | 1.1445470 | 2.3183178 | 8.86E-03 |
| BST2 | 7.1616070 | 1.4420180 | 2.3121942 | 1.07E-02 |
| TAP1 | 609.2425540 | 123.0643770 | 2.3076035 | 2.66E-04 |
| DGKG | 2.1457780 | 0.4411150 | 2.2822741 | 3.56E-03 |
| C1S | 14.4873930 | 2.9926970 | 2.2752799 | 2.64E-03 |
| SECTM1 | 442.5819700 | 92.0589370 | 2.2653150 | 3.57E-04 |
| ACE2 | 2.6171390 | 0.5551320 | 2.2370878 | 6.01E-03 |
| PRDM1 | 3.6154350 | 0.7968390 | 2.1818091 | 5.39E-03 |
| CSF1 | 20.8786560 | 4.6021200 | 2.1816583 | 8.46E-04 |
| UTP14C | 1.2095910 | 0.2684350 | 2.1718746 | 1.70E-02 |
| CYP1B1 | 19.3198660 | 4.2928510 | 2.1700771 | 1.09E-03 |
| HLA-DMB | 4.3506020 | 0.9738790 | 2.1594006 | 6.22E-03 |
| AC008875.3 | 1.9208010 | 0.4305970 | 2.1572979 | 3.19E-02 |
| DNAJA1 | 83.6284480 | 18.9769740 | 2.1397438 | 9.49E-04 |
| HSP90AA2P | 7.8166200 | 1.7893850 | 2.1270811 | 4.68E-03 |
| RGS7 | 3.6462930 | 0.8483860 | 2.1036378 | 2.21E-02 |
| ZNF114 | 15.5944930 | 3.6548680 | 2.0931454 | 8.89E-04 |

| | | | | |
|---|---|---|---|---|
| DDX58 | 21.2645090 | 5.0175230 | 2.0834003 | 1.70E-03 |
| UGT1A3 | 12.5114850 | 2.9565310 | 2.0812757 | 3.16E-03 |
| RIPK2 | 50.3785020 | 12.0040700 | 2.0692846 | 1.70E-03 |
| HELB | 2.3487300 | 0.5621100 | 2.0629565 | 1.74E-02 |
| RTEL1-TNFRSF6B | 3.9943840 | 0.9624870 | 2.0531341 | 9.13E-03 |
| ADAMTS6 | 1.1862530 | 0.2859360 | 2.0526476 | 3.82E-02 |
| PSMB8-AS1 | 13.1968820 | 3.1890560 | 2.0489958 | 7.27E-03 |
| HSPA1A | 759.5011600 | 185.0272830 | 2.0373142 | 1.38E-03 |
| U2AF1 | 82.6732710 | 20.2705480 | 2.0280359 | 4.95E-03 |
| AC016876.3 | 17.4930690 | 4.3032550 | 2.0232832 | 2.24E-03 |
| HSPA1B | 658.8464970 | 162.1849980 | 2.0223020 | 1.50E-03 |
| IFIT5 | 16.3410970 | 4.0314610 | 2.0191302 | 2.47E-03 |
| SAMHD1 | 54.4251440 | 13.4555600 | 2.0160709 | 5.35E-03 |
| EPSTI1 | 25.6881540 | 6.3543960 | 2.0152763 | 1.60E-03 |
| HLA-DPA1 | 1.5336500 | 0.3832080 | 2.0007697 | 2.47E-02 |
| AC126755.6 | 1.1770560 | 0.2945140 | 1.9987748 | 4.53E-02 |
| ANXA2R | 3.9909000 | 1.0002080 | 1.9964141 | 4.24E-02 |
| GNG10 | 34.2170940 | 8.6456180 | 1.9846762 | 3.81E-03 |
| CCL2 | 34.2972150 | 8.6984800 | 1.9792562 | 5.57E-03 |
| RPS24 | 1259.3602290 | 319.8347780 | 1.9772923 | 6.56E-03 |
| PSMA3 | 125.6899410 | 31.9573610 | 1.9756490 | 2.13E-03 |
| CD74 | 32.4766770 | 8.2980340 | 1.9685626 | 4.14E-03 |
| LIPT1 | 5.7001580 | 1.4602390 | 1.9647974 | 4.93E-02 |
| CEACAM6 | 3.9622420 | 1.0215740 | 1.9555233 | 1.66E-02 |
| APOL1 | 45.4720760 | 11.7285340 | 1.9549582 | 2.97E-03 |
| HSP90AA1 | 554.8844600 | 143.5984340 | 1.9501474 | 2.21E-03 |
| NMI | 91.1295090 | 23.9643400 | 1.9270291 | 3.13E-03 |
| DTX3L | 115.1372150 | 30.4576240 | 1.9184789 | 2.67E-03 |
| INPP1 | 192.4491730 | 51.1551210 | 1.9115269 | 3.74E-03 |
| IL15 | 3.7873440 | 1.0187160 | 1.8944346 | 1.24E-02 |
| SOCS3 | 15.0944080 | 4.0634720 | 1.8932293 | 5.70E-03 |
| HS3ST3A1 | 2.1371710 | 0.5775400 | 1.8877096 | 2.55E-02 |
| JAK2 | 3.8146000 | 1.0356530 | 1.8809911 | 2.16E-02 |
| POLR2K | 14.7844070 | 4.0141890 | 1.8808959 | 1.61E-02 |
| AF117829.1 | 4.4800730 | 1.2189640 | 1.8778667 | 2.10E-02 |
| CASP1 | 35.6840590 | 9.7143380 | 1.8770921 | 9.68E-03 |
| IFIT2 | 64.8151780 | 17.6497710 | 1.8766822 | 4.69E-03 |
| SP110 | 59.6184500 | 16.2430000 | 1.8759408 | 1.26E-04 |
| CHMP5 | 31.5554980 | 8.5977620 | 1.8758583 | 5.39E-03 |
| GRAMD2B | 35.0888210 | 9.5757470 | 1.8735545 | 4.22E-03 |
| CETN3 | 8.6101620 | 2.3741160 | 1.8586500 | 3.20E-02 |
| IL1RL1 | 10.6690930 | 2.9580990 | 1.8506953 | 1.00E-02 |
| TOP1 | 130.4019780 | 36.3813900 | 1.8416932 | 4.05E-03 |
| HSPA7 | 9.4870570 | 2.6572030 | 1.8360522 | 1.32E-02 |
| DNAJB4 | 9.5853680 | 2.6976480 | 1.8291317 | 1.63E-03 |
| NUDCD1 | 18.6622430 | 5.2791390 | 1.8217478 | 9.12E-03 |
| RPL7P23 | 25.8350770 | 7.3981960 | 1.8040858 | 1.44E-02 |
| IFI16 | 386.3957210 | 111.0127110 | 1.7993542 | 3.71E-03 |
| APOL6 | 63.7375910 | 18.3215790 | 1.7986006 | 4.96E-03 |
| TXNDC9 | 28.5180870 | 8.2277120 | 1.7933140 | 1.13E-02 |
| UQCRHL | 3.2564600 | 0.9439860 | 1.7864671 | 4.50E-02 |
| NOCT | 8.7112650 | 2.5311090 | 1.7831126 | 1.71E-02 |
| CIBAR1 | 16.5055660 | 4.8290410 | 1.7731440 | 3.22E-02 |
| HSPA6 | 85.6188280 | 25.0709840 | 1.7719095 | 6.18E-03 |
| RSAD2 | 23.0521010 | 6.7675220 | 1.7681987 | 8.05E-03 |
| IFIH1 | 28.1865500 | 8.2820470 | 1.7669476 | 6.32E-03 |
| AC046176.1 | 7.5888800 | 2.2341730 | 1.7641461 | 3.53E-02 |
| TICAM2 | 2.0626860 | 0.6090110 | 1.7599840 | 1.32E-02 |
| ACOT4 | 2.9229760 | 0.8662010 | 1.7546642 | 4.91E-02 |
| ZC3HAV1 | 60.9823530 | 18.1128010 | 1.7513822 | 4.22E-03 |
| SENP3-EIF4A1 | 167.5942380 | 49.8113560 | 1.7504260 | 6.31E-03 |

| | | | | |
|---|---|---|---|---|
| SNRPE | 19.6146530 | 5.8376540 | 1.7484712 | 4.22E-03 |
| COX7B | 28.0039880 | 8.3481440 | 1.7461049 | 6.83E-03 |
| AC004057.1 | 197.1539000 | 58.9016720 | 1.7429418 | 9.15E-03 |
| RTP4 | 9.3356110 | 2.7906740 | 1.7421308 | 2.39E-02 |
| KRT75 | 14.0637080 | 4.2107930 | 1.7398132 | 1.31E-02 |
| DDX60 | 30.3676090 | 9.1134460 | 1.7364647 | 6.94E-03 |
| IL1A | 27.4840890 | 8.2694170 | 1.7327391 | 1.01E-02 |
| SLC2A3 | 6.9949650 | 2.1110060 | 1.7283862 | 1.38E-02 |
| OAS2 | 23.4787980 | 7.0918610 | 1.7271224 | 1.26E-02 |
| RSL24D1 | 98.3228990 | 29.8242700 | 1.7210406 | 8.08E-03 |
| HSPH1 | 71.4224550 | 21.7127820 | 1.7178331 | 5.37E-03 |
| P2RY6 | 16.8754940 | 5.1473900 | 1.7130167 | 1.26E-02 |
| NKX3-1 | 3.5272490 | 1.0771630 | 1.7113068 | 3.20E-02 |
| TEX30 | 10.0290480 | 3.0640960 | 1.7106513 | 3.02E-02 |
| RPAP3 | 28.8077550 | 8.8148160 | 1.7084549 | 4.16E-02 |
| RBMXL1 | 11.8609860 | 3.6450820 | 1.7022008 | 1.33E-02 |
| STX11 | 1.9571110 | 0.6018750 | 1.7011898 | 3.56E-02 |
| NUP35 | 13.3277310 | 4.0995930 | 1.7008786 | 3.88E-02 |
| KIF18A | 13.9244450 | 4.2890390 | 1.6988935 | 1.30E-02 |
| RAB23 | 4.5495620 | 1.4030220 | 1.6971900 | 1.93E-02 |
| EXOC6 | 8.7833260 | 2.7113230 | 1.6957704 | 1.54E-02 |
| PLA2G4A | 6.9938940 | 2.1619780 | 1.6937441 | 2.06E-02 |
| ZUP1 | 7.5324130 | 2.3293880 | 1.6931611 | 2.79E-02 |
| GEMIN2 | 14.8506190 | 4.6304220 | 1.6813075 | 2.87E-02 |
| IL15RA | 52.0235940 | 16.2245540 | 1.6809872 | 2.58E-02 |
| HOXC-AS2 | 4.5447980 | 1.4186860 | 1.6796609 | 4.88E-02 |
| HAT1 | 39.0433920 | 12.3478050 | 1.6608238 | 1.18E-02 |
| RPL26 | 2130.7036130 | 675.8931880 | 1.6564627 | 1.13E-02 |
| OIP5 | 17.0565720 | 5.4126520 | 1.6559202 | 2.25E-02 |
| CYP1A1 | 33.5237580 | 10.6780260 | 1.6505389 | 1.37E-02 |
| SRGAP2D | 11.3626300 | 3.6253280 | 1.6481134 | 3.87E-02 |
| TIA1 | 15.8348630 | 5.0561280 | 1.6469995 | 3.79E-02 |
| C1R | 58.9505730 | 18.9159430 | 1.6399031 | 7.56E-03 |
| NSA2 | 32.8213580 | 10.5369010 | 1.6391843 | 4.70E-02 |
| NMRAL2P | 40.3566280 | 12.9609080 | 1.6386388 | 7.54E-03 |
| AL353807.5 | 9.1966720 | 2.9559440 | 1.6374929 | 3.92E-02 |
| GPR89B | 11.2108790 | 3.6036880 | 1.6373534 | 2.92E-02 |
| TXNRD1 | 144.1141820 | 46.5416910 | 1.6306168 | 1.20E-02 |
| NOC3L | 18.5961860 | 6.0372380 | 1.6230462 | 1.74E-02 |
| EID2 | 13.9764970 | 4.5427380 | 1.6213688 | 2.66E-02 |
| GAS5 | 384.0229190 | 124.8679120 | 1.6207896 | 2.83E-02 |
| SERPINB2 | 497.9999080 | 162.0644990 | 1.6195774 | 1.13E-02 |
| UBE2L6 | 561.9699710 | 183.8239440 | 1.6121683 | 1.33E-02 |
| IFI44 | 14.9288230 | 4.8882510 | 1.6107102 | 2.63E-02 |
| TNFSF10 | 97.4929050 | 31.9253440 | 1.6105951 | 1.12E-02 |
| MPHOSPH6 | 74.7102890 | 24.4882970 | 1.6092145 | 1.48E-02 |
| RPS26P6 | 29.5355530 | 9.6897130 | 1.6079268 | 4.88E-02 |
| AC133555.6 | 4.8319190 | 1.5957810 | 1.5983336 | 3.78E-02 |
| NBN | 19.1522270 | 6.3284150 | 1.5975960 | 1.83E-02 |
| CCDC58 | 40.8704110 | 13.5231220 | 1.5956285 | 2.68E-02 |
| NAMPT | 98.5325160 | 32.6402210 | 1.5939491 | 1.42E-02 |
| DNAJB9 | 15.6621310 | 5.1885300 | 1.5938828 | 2.08E-02 |
| MRPL13 | 23.8455700 | 7.9414920 | 1.5862393 | 2.47E-02 |
| IMPA1 | 7.9449910 | 2.6475590 | 1.5853828 | 1.54E-02 |
| THAP1 | 8.1167240 | 2.7096510 | 1.5827905 | 3.82E-02 |
| DYNLT3 | 11.4390730 | 3.8204730 | 1.5821470 | 2.64E-02 |
| UGT1A4 | 22.3091260 | 7.4727620 | 1.5779205 | 2.10E-02 |
| SMN2 | 27.6149670 | 9.2595920 | 1.5764299 | 2.54E-02 |
| TGDS | 9.3529210 | 3.1464910 | 1.5716732 | 2.44E-02 |
| CPNE8 | 5.0664960 | 1.7053270 | 1.5709399 | 3.58E-02 |
| SCYL3 | 5.3152760 | 1.7919530 | 1.5686118 | 4.62E-02 |

| | | | | |
|---|---|---|---|---|
| BCL6 | 15.7594160 | 5.3200300 | 1.5667078 | 1.99E-02 |
| ANAPC10 | 8.0470670 | 2.7216370 | 1.5639884 | 4.79E-02 |
| DUSP1 | 16.1300810 | 5.4653770 | 1.5613608 | 2.56E-02 |
| CCDC90B | 25.3329220 | 8.6172030 | 1.5557219 | 4.28E-02 |
| SP140L | 15.1572280 | 5.1570810 | 1.5553793 | 2.63E-02 |
| OAS1 | 70.6252370 | 24.0509820 | 1.5540880 | 1.98E-02 |
| MRPL1 | 27.1028100 | 9.2764480 | 1.5467980 | 2.71E-02 |
| RPL7P1 | 292.6794740 | 100.4870910 | 1.5423114 | 1.77E-02 |
| TCEA1P2 | 18.8728600 | 6.4822780 | 1.5417403 | 3.99E-02 |
| SOCS1 | 26.7490750 | 9.2157000 | 1.5373233 | 3.07E-02 |
| ZFAND1 | 97.2351070 | 33.5296750 | 1.5360388 | 1.31E-02 |
| RPL7 | 985.7401120 | 340.3381960 | 1.5342383 | 1.69E-02 |
| ATF3 | 80.5444640 | 27.8270650 | 1.5332967 | 1.73E-02 |
| DYRK4 | 39.3705290 | 13.6135640 | 1.5320713 | 1.97E-02 |
| HSPA8 | 586.0648800 | 202.7845150 | 1.5311129 | 1.74E-02 |
| PDP1 | 88.8801270 | 30.8249280 | 1.5277634 | 1.32E-02 |
| AC034102.1 | 28.5717680 | 9.9150280 | 1.5269016 | 4.06E-02 |
| COQ10B | 11.1334760 | 3.8745510 | 1.5228030 | 3.25E-02 |
| IFT57 | 29.1708950 | 10.1900060 | 1.5173747 | 2.18E-02 |
| GCLM | 31.8347360 | 11.1275410 | 1.5164670 | 1.85E-02 |
| EIF3E | 292.5863950 | 102.3383870 | 1.5155153 | 2.06E-02 |
| SLFN11 | 47.5609780 | 16.6705400 | 1.5124775 | 1.16E-02 |
| TATDN1 | 49.0373610 | 17.2095680 | 1.5106705 | 3.01E-02 |
| SP100 | 48.9831810 | 17.2074720 | 1.5092513 | 1.89E-02 |
| SNAPC1 | 12.8279270 | 4.5112480 | 1.5076895 | 3.09E-02 |
| TRMT11 | 17.6465440 | 6.2120200 | 1.5062513 | 3.99E-02 |
| GBP6 | 40.0732270 | 14.1076600 | 1.5061600 | 2.09E-02 |
| PNPT1 | 16.0585540 | 5.6808680 | 1.4991587 | 2.53E-02 |
| RPS7 | 754.3434450 | 266.8637080 | 1.4991184 | 2.43E-02 |
| TRIM21 | 108.5231480 | 38.4110220 | 1.4984105 | 1.67E-02 |
| MLF1 | 23.6680110 | 8.3872100 | 1.4966756 | 4.91E-02 |
| LINS1 | 9.1054840 | 3.2373380 | 1.4919277 | 1.06E-02 |
| RAB18 | 12.2814550 | 4.3710090 | 1.4904432 | 3.23E-02 |
| CCDC68 | 0.8222770 | 0.2939130 | 1.4842353 | 4.58E-02 |
| MSRB1 | 237.8396000 | 85.0691450 | 1.4832811 | 2.37E-02 |
| CDKN3 | 41.1864620 | 14.7510260 | 1.4813549 | 3.64E-02 |
| FERMT2 | 7.5007310 | 2.6944850 | 1.4770216 | 4.05E-02 |
| EMC2 | 13.7375550 | 4.9461640 | 1.4737433 | 2.64E-02 |
| CD55 | 28.1980860 | 10.1569510 | 1.4731299 | 2.63E-02 |
| OSTM1 | 4.6700730 | 1.6821820 | 1.4731113 | 4.91E-02 |
| PSMA2 | 98.9617540 | 35.6663930 | 1.4723057 | 2.35E-02 |
| C12orf4 | 9.1166040 | 3.2864990 | 1.4719450 | 3.00E-02 |
| ABCG2 | 12.1681610 | 4.3883890 | 1.4713478 | 3.52E-02 |
| C1D | 8.4552300 | 3.0531730 | 1.4695347 | 2.33E-02 |
| HMMR | 30.6028040 | 11.0633040 | 1.4678815 | 2.87E-02 |
| DNAJA4 | 23.8091240 | 8.6107180 | 1.4673091 | 3.63E-02 |
| HMOX1 | 246.7338560 | 89.2808760 | 1.4665326 | 2.29E-02 |
| OSTC | 52.5934030 | 19.0323350 | 1.4664293 | 3.02E-02 |
| RALA | 183.2045140 | 66.3976590 | 1.4642508 | 2.26E-02 |
| SCFD1 | 58.1789210 | 21.0874940 | 1.4641089 | 2.08E-02 |
| COPS4 | 27.7629620 | 10.0687860 | 1.4632717 | 3.23E-02 |
| MX1 | 21.9572530 | 7.9740870 | 1.4613063 | 2.67E-02 |
| BLZF1 | 17.0180890 | 6.1818180 | 1.4609660 | 3.19E-02 |
| TMEM126A | 22.7365490 | 8.2610320 | 1.4606194 | 4.96E-02 |
| LACTB | 36.9952010 | 13.4597690 | 1.4586845 | 3.27E-02 |
| RPS3A | 1465.2781980 | 534.4303590 | 1.4551007 | 2.07E-02 |
| SNX2 | 12.5386690 | 4.5784030 | 1.4534678 | 2.34E-02 |
| SF3B6 | 105.5739210 | 38.5911710 | 1.4519108 | 3.04E-02 |
| KYAT3 | 35.3629420 | 12.9277070 | 1.4517719 | 2.99E-02 |
| PLIN2 | 188.9908750 | 69.1280210 | 1.4509740 | 2.59E-02 |
| RPS3AP26 | 82.5085450 | 30.2745080 | 1.4464400 | 3.17E-02 |

| | | | | |
|---|---|---|---|---|
| USP16 | 17.0800590 | 6.2702320 | 1.4457222 | 3.47E-02 |
| PDCD10 | 59.9241980 | 22.0203500 | 1.4443013 | 2.78E-02 |
| CTNNAL1 | 175.7207180 | 64.8712770 | 1.4376326 | 2.65E-02 |
| KLHDC7B | 33.1675530 | 12.2483760 | 1.4371821 | 2.82E-02 |
| OSGIN2 | 10.4748700 | 3.8686390 | 1.4370343 | 2.12E-02 |
| ZNF267 | 6.6555170 | 2.4626700 | 1.4343274 | 3.46E-02 |
| PPA1 | 251.7202000 | 93.1520310 | 1.4341619 | 2.72E-02 |
| FAM117B | 4.0270870 | 1.4905990 | 1.4338444 | 4.66E-02 |
| LAP3 | 68.7126620 | 25.4701420 | 1.4317690 | 3.40E-02 |
| HSPD1P1 | 17.4020860 | 6.4516040 | 1.4315305 | 4.24E-02 |
| CAPZA2 | 24.6505910 | 9.1416870 | 1.4310899 | 3.01E-02 |
| HIF1A | 69.8071370 | 25.9062500 | 1.4300743 | 2.80E-02 |
| HSPE1 | 82.9815290 | 30.8504870 | 1.4274970 | 3.41E-02 |
| KLF4 | 62.6065250 | 23.2884540 | 1.4266982 | 2.71E-02 |
| UBA6 | 11.9282040 | 4.4388230 | 1.4261277 | 2.73E-02 |
| SPTLC3 | 10.3592340 | 3.8759720 | 1.4182873 | 3.74E-02 |
| RPS7P1 | 71.1137470 | 26.6212080 | 1.4175524 | 3.97E-02 |
| NAE1 | 50.0841600 | 18.7825910 | 1.4149583 | 3.16E-02 |
| BRIX1 | 39.9141770 | 14.9962280 | 1.4123016 | 4.00E-02 |
| NAMPTP1 | 21.5535910 | 8.1053040 | 1.4109901 | 3.78E-02 |
| CIR1 | 27.0212480 | 10.1678820 | 1.4100751 | 3.79E-02 |
| MCUB | 21.2437110 | 8.0020910 | 1.4085869 | 4.29E-02 |
| EIF2S2 | 204.3902590 | 77.2265170 | 1.4041582 | 3.04E-02 |
| COX7C | 108.7474520 | 41.2217750 | 1.3995031 | 4.12E-02 |
| PRR5-ARHGAP8 | 19.4562510 | 7.3824770 | 1.3980569 | 3.08E-02 |
| NUB1 | 141.2012020 | 53.6115610 | 1.3971363 | 3.16E-02 |
| ACTR10 | 22.9870070 | 8.7664750 | 1.3907499 | 3.95E-02 |
| RPL7P9 | 214.1217650 | 82.1055830 | 1.3828792 | 3.54E-02 |
| RPF1 | 33.6179010 | 12.9151980 | 1.3801599 | 4.05E-02 |
| MYOSLID | 38.7395060 | 14.8858050 | 1.3798683 | 3.49E-02 |
| MITD1 | 56.8082200 | 21.8458880 | 1.3787379 | 4.99E-02 |
| TAX1BP1 | 131.8401490 | 50.7252200 | 1.3780147 | 4.15E-02 |
| CWC27 | 21.2943780 | 8.1981260 | 1.3771065 | 4.54E-02 |
| DPM1 | 78.2535550 | 30.3049390 | 1.3686034 | 4.13E-02 |
| IRF2 | 32.1930730 | 12.5417890 | 1.3600071 | 4.51E-02 |
| ATG5 | 14.1661140 | 5.5210990 | 1.3594167 | 4.88E-02 |
| NOP58 | 61.3098640 | 23.8989280 | 1.3591733 | 4.14E-02 |
| FAS | 7.5254320 | 2.9336670 | 1.3590693 | 3.06E-02 |
| DNTTIP2 | 16.3616770 | 6.3820550 | 1.3582277 | 4.41E-02 |
| PRDX3 | 46.5365640 | 18.1910990 | 1.3551320 | 4.56E-02 |
| MMP25-AS1 | 18.3878210 | 7.2182540 | 1.3490287 | 1.30E-02 |
| DAPP1 | 17.1235410 | 6.7277040 | 1.3477949 | 4.81E-02 |
| BACH1 | 11.5459600 | 4.5382000 | 1.3471960 | 4.70E-02 |
| PUM3 | 35.5788920 | 14.0039040 | 1.3451925 | 4.45E-02 |
| CUL2 | 15.6286450 | 6.1552700 | 1.3442987 | 4.04E-02 |
| ABRAXAS1 | 3.6952260 | 1.4577430 | 1.3419262 | 2.46E-02 |
| DNAJC2 | 33.0804820 | 13.0565710 | 1.3412042 | 4.74E-02 |
| ANXA1 | 1294.5944820 | 511.5712590 | 1.3394931 | 3.86E-02 |
| NFE2L2 | 114.2024150 | 45.2110210 | 1.3368468 | 4.03E-02 |
| HIBCH | 22.2274130 | 8.8314850 | 1.3316121 | 4.86E-02 |
| RSRC1 | 24.4553930 | 9.7167880 | 1.3316013 | 4.05E-02 |
| UGDH | 46.5872960 | 18.5853940 | 1.3257673 | 4.36E-02 |
| ZNF277 | 24.6044060 | 9.8178020 | 1.3254447 | 4.45E-02 |
| CASP7 | 35.9834250 | 14.3625770 | 1.3250179 | 4.36E-02 |
| TRIM5 | 45.2199290 | 18.0751020 | 1.3229549 | 4.98E-02 |
| PIR | 115.0908660 | 46.0193600 | 1.3224605 | 4.61E-02 |
| GCH1 | 11.0112560 | 4.4030040 | 1.3224190 | 4.67E-02 |
| ANXA3 | 190.9717410 | 76.4246900 | 1.3212485 | 4.32E-02 |
| SMARCA5 | 20.7833480 | 8.3173080 | 1.3212395 | 4.52E-02 |
| PSMD14 | 81.2932360 | 32.5725440 | 1.3194789 | 4.84E-02 |
| VSNL1 | 39.6416170 | 15.9024930 | 1.3177629 | 4.61E-02 |

| | | | | |
|---|---|---|---|---|
| ZFP36 | 48.9166070 | 19.6446970 | 1.3161844 | 4.75E-02 |
| ME1 | 26.8658640 | 10.7986000 | 1.3149300 | 4.94E-02 |
| RNF19B | 176.4320680 | 71.0612790 | 1.3119772 | 4.42E-02 |
| LYN | 18.6969590 | 7.5380450 | 1.3105413 | 4.49E-02 |
| CFH | 3.9727250 | 1.6082130 | 1.3046704 | 9.49E-03 |
| UACA | 34.8432690 | 14.1204250 | 1.3030965 | 4.64E-02 |
| SSB | 105.0107190 | 42.9058990 | 1.2912887 | 4.99E-02 |
| COPB1 | 111.1491170 | 45.4213290 | 1.2910547 | 4.93E-02 |
| ITGB3BP | 23.6953280 | 9.9254000 | 1.2554055 | 4.52E-02 |
| ERAP2 | 4.2654700 | 1.8294150 | 1.2213223 | 3.03E-02 |
| COPS2 | 52.3110010 | 22.5793720 | 1.2121090 | 4.64E-02 |
| LINC01232 | 5.4630350 | 2.3666910 | 1.2068313 | 4.96E-02 |
| AC087289.3 | 4.9536090 | 2.1726110 | 1.1890501 | 2.70E-02 |
| TPT1 | 721.4099120 | 318.1689150 | 1.1810264 | 4.58E-02 |
| B2M | 1175.3867190 | 527.3269040 | 1.1563660 | 4.13E-02 |
| SOD2 | 89.2488100 | 40.8259700 | 1.1283458 | 3.60E-02 |
| ATP5F1E | 34.7066880 | 15.8843700 | 1.1276058 | 4.24E-02 |
| MMAA | 4.3168570 | 2.0723250 | 1.0587310 | 3.24E-02 |
| TMEM141 | 338.9603880 | 682.9733890 | -1.0107127 | 4.99E-02 |
| RIPK4 | 87.1063920 | 177.0844270 | -1.0235869 | 4.77E-02 |
| CKB | 85.8720550 | 176.1342470 | -1.0364148 | 4.07E-02 |
| DUSP9 | 30.0361160 | 62.2874950 | -1.0522443 | 4.54E-02 |
| KRT15 | 2363.4375000 | 4910.6982420 | -1.0550415 | 2.74E-02 |
| NOTCH3 | 2.3751820 | 4.9570430 | -1.0614417 | 4.81E-02 |
| PARD6A | 27.7617680 | 58.2109640 | -1.0681915 | 4.65E-02 |
| HMCN2 | 4.4029540 | 9.2471050 | -1.0705300 | 3.09E-02 |
| SREBF1 | 89.5619200 | 189.4192200 | -1.0806254 | 3.80E-02 |
| MIEF2 | 26.3381350 | 55.8317340 | -1.0839322 | 3.29E-02 |
| FAT2 | 22.6421450 | 48.0464060 | -1.0854179 | 3.71E-02 |
| CARMN | 0.1700150 | 0.3614070 | -1.0879624 | 1.99E-02 |
| SLC27A5 | 4.6636110 | 9.9232150 | -1.0893602 | 4.00E-02 |
| TENT5B | 9.6123830 | 20.4626830 | -1.0900293 | 4.64E-02 |
| ARSI | 66.3368610 | 144.7428590 | -1.1256095 | 3.19E-02 |
| AOC1 | 3.1666730 | 6.9675980 | -1.1376935 | 4.93E-02 |
| KCNN4 | 32.1419910 | 71.7368240 | -1.1582546 | 2.89E-02 |
| CEBPA | 11.0560870 | 24.7435860 | -1.1622137 | 3.30E-02 |
| USH1G | 4.2643210 | 9.5719010 | -1.1664894 | 2.82E-02 |
| AHNAK2 | 45.9756890 | 103.6410520 | -1.1726525 | 2.56E-02 |
| TGM1 | 108.4678960 | 245.9682160 | -1.1812038 | 2.33E-02 |
| THEM6 | 60.2830770 | 137.1904300 | -1.1863549 | 2.51E-02 |
| PAQR7 | 53.5424920 | 122.0462880 | -1.1886722 | 2.47E-02 |
| CELSR3 | 0.9860270 | 2.2481990 | -1.1890707 | 2.59E-02 |
| JPH2 | 2.5546940 | 5.8729790 | -1.2009420 | 3.55E-02 |
| SLX1A-SULT1A3 | 12.9097390 | 29.6850950 | -1.2012789 | 3.05E-02 |
| NBPF19 | 3.0584770 | 7.1211100 | -1.2192887 | 2.51E-02 |
| ST6GALNAC1 | 3.0448180 | 7.1090660 | -1.2233040 | 3.28E-02 |
| CDC42EP2 | 12.6775320 | 29.6630950 | -1.2263952 | 2.63E-02 |
| ATP6V1B1 | 8.3204440 | 19.6086790 | -1.2367599 | 2.80E-02 |
| KRT19 | 5391.8588870 | 12773.7402340 | -1.2443264 | 2.06E-02 |
| AC007325.2 | 15.1331560 | 35.8650360 | -1.2448652 | 2.24E-02 |
| HES2 | 19.5708470 | 46.4113040 | -1.2457700 | 2.03E-02 |
| RASA4 | 9.5319100 | 22.7068920 | -1.2522930 | 1.70E-02 |
| VILL | 4.9591080 | 11.8899980 | -1.2615959 | 2.86E-02 |
| KIAA0513 | 1.8669700 | 4.4934730 | -1.2671322 | 4.01E-02 |
| UPK3B | 23.9568880 | 57.9700580 | -1.2748674 | 2.76E-02 |
| RBBP8NL | 3.2689830 | 7.9129240 | -1.2753690 | 3.14E-02 |
| EPHB3 | 37.7799680 | 93.7350770 | -1.3109675 | 1.45E-02 |
| TINCR | 15.0547030 | 37.4845770 | -1.3160829 | 1.40E-02 |
| CCDC88B | 3.5663230 | 8.9214570 | -1.3228420 | 4.16E-02 |
| RUNDC3A | 4.0130510 | 10.0607370 | -1.3259646 | 3.54E-02 |
| EVPLL | 2.0794040 | 5.2350060 | -1.3320211 | 4.63E-02 |

| | | | | |
|---|---|---|---|---|
| EXOC3L4 | 10.2702430 | 26.2022230 | -1.3512189 | 2.95E-02 |
| FAM83E | 3.3595520 | 8.6009860 | -1.3562332 | 3.83E-02 |
| MAB21L4 | 10.7155490 | 27.4661410 | -1.3579485 | 1.49E-02 |
| BARX2 | 8.0681900 | 21.0410840 | -1.3828921 | 1.59E-02 |
| PRSS36 | 1.0250800 | 2.6823700 | -1.3877717 | 4.90E-02 |
| GGT6 | 2.6318360 | 6.9273320 | -1.3962302 | 2.40E-02 |
| AC073957.3 | 0.6059370 | 1.6019550 | -1.4025939 | 3.44E-02 |
| GDPD2 | 1.7812400 | 4.8842240 | -1.4552475 | 3.06E-02 |
| RPL9P7 | 193.5504610 | 539.5046390 | -1.4789256 | 6.65E-03 |
| AC106782.1 | 4.3482190 | 12.1358670 | -1.4807807 | 1.93E-02 |
| BMF | 3.3078360 | 9.2814170 | -1.4884574 | 1.31E-02 |
| TMEM265 | 8.4400710 | 23.9025940 | -1.5018402 | 9.55E-03 |
| AC243919.1 | 207.4583280 | 591.7537840 | -1.5121755 | 5.56E-03 |
| CFD | 16.3265060 | 47.2398640 | -1.5327887 | 9.21E-03 |
| ANKRD2 | 6.1032360 | 17.7572290 | -1.5407602 | 9.73E-03 |
| CIDEB | 2.1340830 | 6.2464690 | -1.5494246 | 2.04E-02 |
| TEN1 | 13.6496830 | 40.0355530 | -1.5524143 | 7.40E-03 |
| LY6G6C | 2.6253240 | 7.7153440 | -1.5552350 | 4.43E-02 |
| AC004951.2 | 5.5458140 | 16.4319690 | -1.5670342 | 1.71E-02 |
| COL5A3 | 1.5975200 | 4.7484540 | -1.5716239 | 8.40E-03 |
| FLG | 3.4548280 | 10.2740350 | -1.5723171 | 4.69E-03 |
| ENTPD8 | 1.2881840 | 3.8756940 | -1.5891160 | 1.76E-02 |
| WFDC5 | 1.8047180 | 5.4331250 | -1.5900088 | 4.40E-02 |
| FOXN1 | 0.7269540 | 2.2152950 | -1.6075628 | 4.45E-02 |
| PRRX2 | 2.6406490 | 8.1047080 | -1.6178677 | 1.83E-02 |
| ZBED6 | 2.1274770 | 6.5360120 | -1.6192671 | 4.20E-03 |
| AC108488.2 | 0.6410720 | 1.9990830 | -1.6407801 | 3.80E-02 |
| PIK3IP1 | 13.7195280 | 43.0640750 | -1.6502540 | 3.41E-03 |
| MMP13 | 0.9063850 | 2.8509260 | -1.6532347 | 2.45E-02 |
| TJP3 | 36.6339760 | 115.7810520 | -1.6601450 | 2.88E-03 |
| PRODH | 12.6120350 | 40.5841100 | -1.6861139 | 6.63E-03 |
| RNF225 | 1.3360310 | 4.3803580 | -1.7130953 | 4.58E-02 |
| VPS37D | 1.0139930 | 3.3286300 | -1.7148808 | 3.34E-02 |
| RASGRP2 | 0.4880200 | 1.6563960 | -1.7630354 | 3.88E-02 |
| BGN | 0.5971330 | 2.0354870 | -1.7692498 | 3.54E-02 |
| PP7080 | 1.7199640 | 5.9085790 | -1.7804328 | 1.09E-02 |
| PSAPL1 | 3.8557610 | 13.2757120 | -1.7837017 | 2.08E-03 |
| NRG2 | 0.8465280 | 2.9717860 | -1.8117005 | 4.86E-02 |
| JMJD7 | 24.5091880 | 86.7813490 | -1.8240623 | 6.94E-04 |
| KCNK7 | 1.2281740 | 4.4238480 | -1.8487868 | 2.56E-02 |
| AC138696.1 | 0.9502100 | 3.4301500 | -1.8519534 | 9.14E-03 |
| AQP10 | 1.7875770 | 6.5784640 | -1.8797454 | 7.71E-03 |
| FGF1 | 0.2262630 | 0.8421330 | -1.8960474 | 1.66E-02 |
| BCL2L2-PABPN1 | 1.8105240 | 6.8084970 | -1.9109291 | 3.29E-03 |
| MUC16 | 1.7309000 | 6.5626310 | -1.9227519 | 3.39E-04 |
| AC132872.5 | 0.4480720 | 1.7511610 | -1.9665092 | 2.30E-02 |
| HS3ST6 | 0.9456700 | 3.9765390 | -2.0721046 | 1.68E-02 |
| LOXL4 | 0.1953950 | 0.8310010 | -2.0884567 | 4.17E-02 |
| CALML5 | 0.9246430 | 4.0000300 | -2.1130425 | 2.77E-02 |
| MSANTD3-TMEFF1 | 0.4594060 | 2.1339780 | -2.2157037 | 1.39E-02 |
| FOXJ1 | 0.3434980 | 1.6574850 | -2.2706222 | 1.36E-02 |
| COL9A2 | 1.1732580 | 5.7719910 | -2.2985488 | 4.17E-03 |
| PTGES3L-AARSD1 | 0.5126490 | 2.5728750 | -2.3273381 | 2.14E-03 |
| PCDHGB1 | 0.0930410 | 0.4774190 | -2.3593175 | 1.59E-02 |
| TPPP3 | 2.6757170 | 14.4194140 | -2.4300151 | 9.22E-04 |
| KRT79 | 0.3461970 | 1.9418810 | -2.4877897 | 6.11E-03 |
| TMEM256-PLSCR3 | 3.6706530 | 21.3969350 | -2.5432955 | 2.54E-07 |
| AC011511.1 | 0.6295480 | 3.7815980 | -2.5866077 | 8.90E-03 |
| ABCF2-H2BE1 | 0.1407840 | 0.8612730 | -2.6129872 | 4.51E-02 |
| NOTCH2NLC | 1.1094090 | 6.8958950 | -2.6359465 | 2.95E-04 |
| RASA4CP | 1.3136460 | 8.3278500 | -2.6643675 | 2.51E-03 |

| | | | | |
|---|---|---|---|---|
| VWF | 0.1482500 | 1.0041650 | -2.7598923 | 4.79E-04 |
| AL162151.2 | 128.9651790 | 969.2300420 | -2.9098575 | 1.61E-06 |
| AL358113.1 | 0.3659750 | 2.8633740 | -2.9678991 | 1.21E-06 |
| SSTR5 | 0.2010160 | 1.5925270 | -2.9859356 | 2.11E-02 |
| AL669830.2 | 2.0968490 | 16.9930590 | -3.0186507 | 1.02E-06 |
| AC002310.4 | 0.1248910 | 1.0715040 | -3.1008958 | 3.73E-02 |
| MGAT3 | 0.1742430 | 2.0470360 | -3.5543639 | 1.91E-04 |
| AC023509.1 | 1.6360560 | 28.1013340 | -4.1023446 | 2.74E-09 |
| NOXO1 | 0.1481640 | 2.7585860 | -4.2186621 | 6.69E-03 |
| AC021092.1 | 0.0146490 | 0.2738920 | -4.2247330 | 2.52E-02 |
| AC005833.1 | 0.0329060 | 0.6266890 | -4.2513271 | 4.36E-03 |
| POC1B-GALNT4 | 0.0615190 | 1.2106290 | -4.2985810 | 3.15E-07 |
| UPK3BP1 | 0.2116460 | 4.4388690 | -4.3904670 | 2.66E-03 |
| AC004922.1 | 0.4258470 | 10.7653270 | -4.6599131 | 2.16E-10 |
| AC005538.2 | 0.0199990 | 0.5389740 | -4.7522159 | 4.79E-02 |
| ZG16 | 0.0001000 | 0.2312110 | -11.1749943 | 3.75E-02 |
| LRRC37A16P | 0.0001000 | 0.3494120 | -11.7707134 | 3.28E-03 |
| AC087721.2 | 0.0001000 | 0.4367440 | -12.0925722 | 1.01E-02 |
| UGT1A5 | 0.0001000 | 0.4644750 | -12.1813852 | 7.85E-03 |
| AC067968.1 | 0.0001000 | 0.5403020 | -12.3995503 | 1.84E-04 |
| THEM5 | 0.0001000 | 0.6073420 | -12.5682934 | 4.29E-02 |
| AL163636.2 | 0.0001000 | 0.9322840 | -13.1865538 | 2.28E-03 |
| AC104109.3 | 0.0001000 | 1.3652140 | -13.7368395 | 4.91E-02 |
| AC010507.1 | 0.0001000 | 1.8483050 | -14.1739152 | 3.75E-02 |
| AL136295.1 | 0.0001000 | 2.3300120 | -14.5080498 | 1.06E-09 |
| Z97353.1 | 0.0001000 | 2.4785500 | -14.5972087 | 1.79E-03 |
| AC139769.2 | 0.0001000 | 42.9419940 | -18.7120297 | 1.14E-28 |
