# Supplementary material for: Advanced whole transcriptome sequencing and artificial intelligence/machine learning (AI/ML) in imiquimod-induced psoriasis-like inflammation of human keratinocytes
Source: Biomedicine (Taipei). 2024 Dec 1;14(4):36–50. doi: 10.37796/2211-8039.1468 (PMC11703395; doi:10.37796/2211-8039.1468)
Supplement: Supplementary file 2 [file SupplementaryTableS2.pdf]

Supplementary Table S2: Raw results of machine learning (ML) disease pathways prediction analysis							
**My Pathways**	**-Log(p-value)**	**Ratio**	**Down-regulated**	**No change**	**Up-regulated**	**No overlap with dataset**	**Molecules**
Multiple Sclerosis	6.38	0.195	0/41 (0%)	0/41 (0%)	8/41 (20%)	33/41 (80%)	FAS,MX1,OAS1,RSAD2,SAMHD1,TAP1,TICAM2,WARS1
Mixed hematopoietic and lymphoid cancer	5.9	0.17	0/47 (0%)	0/47 (0%)	8/47 (17%)	39/47 (83%)	CCL2,CYP1A1,CYP1B1,IFT57,NMI,SERPINB2,TNFSF10,XAF1
Lymphoproliferative malignancy	5.9	0.17	0/47 (0%)	0/47 (0%)	8/47 (17%)	39/47 (83%)	CCL2,CYP1A1,CYP1B1,IFT57,NMI,SERPINB2,TNFSF10,XAF1
Polycythemia vera	4.77	0.146	0/48 (0%)	0/48 (0%)	7/48 (15%)	41/48 (85%)	IRF1,JAK2,NLRC5,STAT1,TNFSF10,UBE2L6,XAF1
Glomerulonephritis	4.65	0.176	0/34 (0%)	0/34 (0%)	6/34 (18%)	28/34 (82%)	HMOX1,IFIH1,IL6,LYN,NFE2L2,SOCS1
Alopecia areata	4.5	0.167	0/36 (0%)	0/36 (0%)	6/36 (17%)	30/36 (83%)	APOL6,BCL6,IL15,IRF1,JAK2,STAT1
Tularemia	4.43	0.162	0/37 (0%)	0/37 (0%)	6/37 (16%)	31/37 (84%)	CASP1,CCL2,IL1A,IL6,IRF1,STAT1
Thymus gland tumor	4.37	0.127	1/55 (2%)	0/55 (0%)	6/55 (11%)	48/55 (87%)	CASP1,HSPE1,IFT57,NMI,SERPINB2,SSTR5,XAF1
Chronic myeloproliferative neoplasm	4.32	0.125	1/56 (2%)	0/56 (0%)	6/56 (11%)	49/56 (88%)	EIF2S2,EMC2,IFIH1,IRF1,JAK2,NOTCH3,RIGI
Chronic malignant hematological neoplasm	4.32	0.125	1/56 (2%)	0/56 (0%)	6/56 (11%)	49/56 (88%)	EIF2S2,EMC2,IFIH1,IRF1,MLF1,NOTCH3,RIGI
Lymphoma	4.27	0.123	0/57 (0%)	0/57 (0%)	7/57 (12%)	50/57 (88%)	CCL2,IFT57,NBN,NMI,SERPINB2,TNFSF10,XAF1
Relapsing-remitting multiple sclerosis	4.22	0.121	0/58 (0%)	0/58 (0%)	7/58 (12%)	51/58 (88%)	EPSTI1,OAS1,RSAD2,SAMHD1,TAP1,TICAM2,WARS1
Neuromuscular disease	4.17	0.146	0/41 (0%)	0/41 (0%)	6/41 (15%)	35/41 (85%)	CTSS,EPSTI1,OAS1,PDCD1LG2,STAT1,WARS1
Anthrax	4.06	0.185	0/27 (0%)	0/27 (0%)	5/27 (19%)	22/27 (81%)	CASP1,CXCL10,IDO1,NLRC5,STAT1
Septic shock	3.99	0.136	0/44 (0%)	0/44 (0%)	6/44 (14%)	38/44 (86%)	ATF3,ICAM1,IL1A,IRF1,RIPK2,STAT1
Ulcer	3.99	0.136	0/44 (0%)	0/44 (0%)	6/44 (14%)	38/44 (86%)	APOL6,CASP1,CXCL10,PLA2G4A,SOCS1,STAT1
Bacterial peritonitis	3.98	0.179	0/28 (0%)	0/28 (0%)	5/28 (18%)	23/28 (82%)	ATF3,CCL2,IL6,P2RY6,RIPK2
Macrophage activation syndrome or hemophagocytic lymphohistiocytosis	3.94	0.133	0/45 (0%)	0/45 (0%)	6/45 (13%)	39/45 (87%)	CCL2,IL1A,IL6,JAK2,STAT1,STX11
Hemophagocytic lymphohistiocytosis	3.94	0.133	0/45 (0%)	0/45 (0%)	6/45 (13%)	39/45 (87%)	CCL2,IL1A,IL6,JAK2,STAT1,STX11
Respiratory distress syndrome	3.9	0.172	0/29 (0%)	0/29 (0%)	5/29 (17%)	24/29 (83%)	JAK2,NFE2L2,NMI,RIGI,TNFRSF1B
Hemophagocytic syndrome	3.88	0.13	0/46 (0%)	0/46 (0%)	6/46 (13%)	40/46 (87%)	CCL2,IL1A,IL6,JAK2,STAT1,STX11
Small-cell carcinoma	3.78	0.125	0/48 (0%)	0/48 (0%)	6/48 (13%)	42/48 (88%)	B2M,CD274,EIF2S2,MLF1,SOD2,TOP1
Uveitis	3.76	0.161	0/31 (0%)	0/31 (0%)	5/31 (16%)	26/31 (84%)	ANXA1,IRF1,PDCD1LG2,STAT1,TNFRSF1B
Myasthenia gravis	3.76	0.161	0/31 (0%)	0/31 (0%)	5/31 (16%)	26/31 (84%)	CD55,CTSS,IL1A,PDCD1LG2,STAT1
Shock Response	3.73	0.122	0/49 (0%)	0/49 (0%)	6/49 (12%)	43/49 (88%)	ATF3,ICAM1,IRF1,RIPK2,STAT1,TNFRSF1B
Development of urinary tract tumor	3.68	0.12	0/50 (0%)	0/50 (0%)	6/50 (12%)	44/50 (88%)	ATF3,HIF1A,HSPE1,KLF4,NAE1,NFE2L2
Chronic myeloid leukemia	3.63	0.118	1/51 (2%)	0/51 (0%)	5/51 (10%)	45/51 (88%)	EIF2S2,EMC2,IFIH1,NOTCH3,RIGI,SOCS3
Infection by Mycobacterium bovis	3.56	0.147	0/34 (0%)	0/34 (0%)	5/34 (15%)	29/34 (85%)	IL15,IL1A,IRF1,PDCD1LG2,STAT1
Mediastinal neoplasm	3.54	0.113	0/53 (0%)	0/53 (0%)	6/53 (11%)	47/53 (89%)	CASP1,HSPE1,IFT57,NMI,SERPINB2,XAF1
Growth of tumor	3.5	0.143	0/35 (0%)	0/35 (0%)	5/35 (14%)	30/35 (86%)	CSF1,HIF1A,IL6,NMI,SLC2A3
Insulin-dependent diabetes mellitus	3.39	0.135	0/37 (0%)	0/37 (0%)	5/37 (14%)	32/37 (86%)	B2M,CD274,CIITA,PARP9,PDCD1LG2
Severe liver disease	3.39	0.135	2/37 (5%)	0/37 (0%)	3/37 (8%)	32/37 (86%)	EIF2S2,IFIT3,PSMD14,RIPK4,TINCR
Neuroendocrine carcinoma	3.36	0.105	0/57 (0%)	0/57 (0%)	6/57 (11%)	51/57 (89%)	B2M,CD274,EIF2S2,NMI,SOD2,TOP1
Hypertrophy	3.33	0.132	2/38 (5%)	0/38 (0%)	3/38 (8%)	33/38 (87%)	EDNRA,IL6,NOTCH3,SERPINB2,SREBF1
Acute respiratory disorder	3.28	0.128	0/39 (0%)	0/39 (0%)	5/39 (13%)	34/39 (87%)	ACE2,CASP1,DUSP1,JAK2,RIPK2
Malignant neuroendocrine neoplasm	3.24	0.1	0/60 (0%)	0/60 (0%)	6/60 (10%)	54/60 (90%)	B2M,CD274,EIF2S2,NMI,SOD2,TNFSF10
Leukopenia	3.23	0.125	0/40 (0%)	0/40 (0%)	5/40 (13%)	35/40 (88%)	HSPH1,IL1A,IL6,SOCS1,STAT1
Small cell lung carcinoma	3.18	0.122	0/41 (0%)	0/41 (0%)	5/41 (12%)	36/41 (88%)	B2M,CD274,EIF2S2,NMI,TOP1
Oxidative stress	3.18	0.122	0/41 (0%)	0/41 (0%)	5/41 (12%)	36/41 (88%)	GCLM,HMOX1,NFE2L2,SOD2,UGDH
Relapsed multiple sclerosis	3.16	0.0968	0/62 (0%)	0/62 (0%)	6/62 (10%)	56/62 (90%)	EPSTI1,IFIH1,RSAD2,SAMHD1,TAP1,WARS1
Lung neuroendocrine carcinoma	3.13	0.119	0/42 (0%)	0/42 (0%)	5/42 (12%)	37/42 (88%)	B2M,CD274,EIF2S2,NMI,TOP1
Lymphatic system tumor	3.13	0.119	0/42 (0%)	0/42 (0%)	5/42 (12%)	37/42 (88%)	CCL2,CYP1B1,IRF1,SERPINB2,XAF1
Neuroendocrine lung cancer	3.13	0.119	0/42 (0%)	0/42 (0%)	5/42 (12%)	37/42 (88%)	B2M,CD274,EIF2S2,NMI,TOP1
Thoracic neuroendocrine neoplasm	3.13	0.119	0/42 (0%)	0/42 (0%)	5/42 (12%)	37/42 (88%)	B2M,CD274,EIF2S2,NMI,TOP1
Ovarian tumor	3.09	0.0938	0/64 (0%)	0/64 (0%)	6/64 (9%)	58/64 (91%)	CYP1A1,CYP1B1,DUSP1,EDNRA,MLF1,SLFN11
Rheumatic Disease	3.08	0.116	0/43 (0%)	0/43 (0%)	5/43 (12%)	38/43 (88%)	CASP1,DUSP1,IL1A,IL6,RIPK2
Rheumatoid arthritis	3.08	0.116	0/43 (0%)	0/43 (0%)	5/43 (12%)	38/43 (88%)	CIITA,IL15,IL1A,IL6,TNFRSF1B
Bronchopathy	3.08	0.116	0/43 (0%)	0/43 (0%)	5/43 (12%)	38/43 (88%)	CCL2,IRF1,STAT1,TNFSF10,XAF1
Protozoan infection	3.03	0.114	0/44 (0%)	0/44 (0%)	5/44 (11%)	39/44 (89%)	IL1A,IRF1,OAS1,TNFRSF1B,TNFSF10
Chronic brain damage	3.02	0.154	0/26 (0%)	0/26 (0%)	4/26 (15%)	22/26 (85%)	BATF2,IRF1,SOCS1,STAT1
Thymic epithelial tumor	3.02	0.154	1/26 (4%)	0/26 (0%)	3/26 (12%)	22/26 (85%)	CASP1,HSPH1,SSTR5,XAF1
Development of adenocarcinoma	2.95	0.109	1/46 (2%)	0/46 (0%)	4/46 (9%)	41/46 (89%)	CEBPA,MLF1,NBN,NMI,STAT1
Failure of liver	2.95	0.109	1/46 (2%)	0/46 (0%)	4/46 (9%)	41/46 (89%)	CEBPA,DUSP1,FAS,HMOX1,IL6
Cerebellar lesion	2.9	0.106	0/47 (0%)	0/47 (0%)	5/47 (11%)	42/47 (89%)	FERMT2,HSP90AA1,NBN,RAB23,SLFN11
Infection by Influenza A virus	2.9	0.106	0/47 (0%)	0/47 (0%)	5/47 (11%)	42/47 (89%)	IL6,IRF1,RIGI,SOCS1,STAT1
Polycythemia	2.9	0.106	0/47 (0%)	0/47 (0%)	5/47 (11%)	42/47 (89%)	IL15,IL1A,IL6,JAK2,ZFP36
Acute lung injury	2.86	0.104	0/48 (0%)	0/48 (0%)	5/48 (10%)	43/48 (90%)	ACE2,CASP1,IL6,JAK2,XAF1
Metastatic melanoma	2.86	0.104	0/48 (0%)	0/48 (0%)	5/48 (10%)	43/48 (90%)	CASP1,CUL2,MLF1,TIA1,XAF1
Lymphoreticular neoplasm	2.86	0.104	1/48 (2%)	0/48 (0%)	4/48 (8%)	43/48 (90%)	CEBPA,CYP1B1,MLF1,RIGI,TNFSF10

Osteopetrosis	2.82	0.102	1/49 (2%)	0/49 (0%)	4/49 (8%)	44/49 (90%)	CEBPA,CSF1,DUSP1,IL1A,OSTM1
Essential thrombocythemia	2.82	0.102	0/49 (0%)	0/49 (0%)	5/49 (10%)	44/49 (90%)	IRF1,JAK2,NLRC5,STAT1,UBE2L6
High bone mass disease	2.82	0.102	1/49 (2%)	0/49 (0%)	4/49 (8%)	44/49 (90%)	CEBPA,CSF1,DUSP1,IL1A,OSTM1
Malignant neoplasm of cerebellum	2.82	0.102	0/49 (0%)	0/49 (0%)	5/49 (10%)	44/49 (90%)	FERMT2,HSP90AA1,NBN,RAB23,SLFN11
Pancreaticobiliary carcinoma	2.82	0.102	1/49 (2%)	0/49 (0%)	4/49 (8%)	44/49 (90%)	CD274,IL6,TNFSF10,TOP1,VWF
Hereditary malignant hematological system tumor	2.82	0.102	1/49 (2%)	0/49 (0%)	4/49 (8%)	44/49 (90%)	CEBPA,IRF1,NLRC5,STAT1,UBE2L6
Medulloblastoma	2.78	0.1	0/50 (0%)	0/50 (0%)	5/50 (10%)	45/50 (90%)	FERMT2,HSP90AA1,NBN,RAB23,SLFN11
Cerebellar neoplasm	2.78	0.1	0/50 (0%)	0/50 (0%)	5/50 (10%)	45/50 (90%)	FERMT2,HSP90AA1,NBN,RAB23,SLFN11
Development of renal tumor	2.7	0.0962	0/52 (0%)	0/52 (0%)	5/52 (10%)	47/52 (90%)	ATF3,HIF1A,HSPE1,NAE1,SLC2A3
Chronic lymphocytic leukemia	2.7	0.0962	1/52 (2%)	0/52 (0%)	4/52 (8%)	47/52 (90%)	CEBPA,HAT1,IRF9,KLF4,SLC2A3
Allergic contact dermatitis	2.7	0.0962	0/52 (0%)	0/52 (0%)	5/52 (10%)	47/52 (90%)	B2M,CIITA,ICAM1,NLRC5,PARP9
Attention Deficit Disorders	2.67	0.125	0/32 (0%)	0/32 (0%)	4/32 (13%)	28/32 (88%)	GBP2,IFIT2,IRF9,ZC3HAV1
Severe hepatic system disorder	2.67	0.125	2/32 (6%)	0/32 (0%)	2/32 (6%)	28/32 (88%)	IFIT3,NBN,RIPK4,TINCR
Short attention span	2.67	0.125	0/32 (0%)	0/32 (0%)	4/32 (13%)	28/32 (88%)	GBP2,IFIT2,IRF9,ZC3HAV1
Neck neoplasm	2.63	0.0926	0/54 (0%)	0/54 (0%)	5/54 (9%)	49/54 (91%)	HSPE1,NBN,OAS1,SERPINB2,SNRPE
Intestinal adenocarcinoma	2.63	0.0926	1/54 (2%)	0/54 (0%)	4/54 (7%)	49/54 (91%)	B2M,DNAJA4,HSPE1,NBN,PARD6A
Ankylosis	2.62	0.121	0/33 (0%)	0/33 (0%)	4/33 (12%)	29/33 (88%)	CIITA,NLRC5,PARP9,STAT1
DNA repair-deficiency disorder	2.62	0.121	0/33 (0%)	0/33 (0%)	4/33 (12%)	29/33 (88%)	BCL6,HSPH1,NBN,SLC2A3
Soft tissue sarcoma	2.6	0.0909	0/55 (0%)	0/55 (0%)	5/55 (9%)	50/55 (91%)	ATF3,HMOX1,MLF1,NMI,WARS1
Malignant neoplasm of soft connective tissue	2.6	0.0909	0/55 (0%)	0/55 (0%)	5/55 (9%)	50/55 (91%)	ATF3,HMOX1,MLF1,NMI,WARS1
Adenocarcinoma	2.57	0.118	1/34 (3%)	0/34 (0%)	3/34 (9%)	30/34 (88%)	CEBPA,NBN,NMI,TNFSF10
Lymphoproliferative disorder	2.57	0.118	0/34 (0%)	0/34 (0%)	4/34 (12%)	30/34 (88%)	CCL2,CYP1A1,FAS,TNFSF10
Tongue disease	2.57	0.118	0/34 (0%)	0/34 (0%)	4/34 (12%)	30/34 (88%)	HSP90AA1,IFIH1,PARP9,RIGI
Large-cell diffuse lymphoma	2.56	0.0893	2/56 (4%)	0/56 (0%)	3/56 (5%)	51/56 (91%)	BCL6,BMF,KLF4,NOC3L,RIPK4
Diffuse aggressive lymphoma	2.56	0.0893	2/56 (4%)	0/56 (0%)	3/56 (5%)	51/56 (91%)	BCL6,BMF,KLF4,NOC3L,RIPK4
Diffuse large B-cell lymphoma	2.56	0.0893	2/56 (4%)	0/56 (0%)	3/56 (5%)	51/56 (91%)	BCL6,BMF,KLF4,NOC3L,RIPK4
Large cell non-Hodgkin lymphoma	2.56	0.0893	2/56 (4%)	0/56 (0%)	3/56 (5%)	51/56 (91%)	BCL6,BMF,KLF4,NOC3L,RIPK4
Aggressive large-cell diffuse lymphoma	2.56	0.0893	2/56 (4%)	0/56 (0%)	3/56 (5%)	51/56 (91%)	BCL6,BMF,KLF4,NOC3L,RIPK4
Chronic form of lymphocytic leukemia	2.53	0.0877	0/57 (0%)	0/57 (0%)	5/57 (9%)	52/57 (91%)	IRF1,KLF4,NLRC5,STAT1,TNFSF10
Tubular adenocarcinoma	2.53	0.0877	0/57 (0%)	0/57 (0%)	5/57 (9%)	52/57 (91%)	CD274,NLRC5,OAS1,STAT1,TAP1
Myasthenia	2.53	0.114	0/35 (0%)	0/35 (0%)	4/35 (11%)	31/35 (89%)	CTSS,IL1A,PDCD1LG2,STAT1
Pulmonary hypertensive arterial disease	2.53	0.114	0/35 (0%)	0/35 (0%)	4/35 (11%)	31/35 (89%)	EDNRA,HSPE1,SERPINB2,TNFSF10
Articular rigidity	2.53	0.114	0/35 (0%)	0/35 (0%)	4/35 (11%)	31/35 (89%)	CIITA,NLRC5,PARP9,STAT1
Myasthenic syndrome	2.53	0.114	0/35 (0%)	0/35 (0%)	4/35 (11%)	31/35 (89%)	CTSS,IL1A,PDCD1LG2,STAT1
Pulmonary arterial hypertension or pulmonary veno-occlusive disease	2.53	0.114	0/35 (0%)	0/35 (0%)	4/35 (11%)	31/35 (89%)	EDNRA,HSPE1,SERPINB2,TNFSF10
Primitive neuroectodermal tumor	2.49	0.0862	0/58 (0%)	0/58 (0%)	5/58 (9%)	53/58 (91%)	FERMT2,NBN,RAB23,TNFSF10,TOP1
Neuroectodermal tumor	2.49	0.0862	0/58 (0%)	0/58 (0%)	5/58 (9%)	53/58 (91%)	FERMT2,NBN,RAB23,TNFSF10,TOP1
Primitive nervous system tumor	2.49	0.0862	0/58 (0%)	0/58 (0%)	5/58 (9%)	53/58 (91%)	FERMT2,NBN,RAB23,TNFSF10,TOP1
Myocarditis	2.48	0.111	0/36 (0%)	0/36 (0%)	4/36 (11%)	32/36 (89%)	BCL6,CD274,IL1A,STAT1
Plasmacytoma	2.46	0.0847	1/59 (2%)	0/59 (0%)	4/59 (7%)	54/59 (92%)	HSP90AA1,IL6,KLF4,MUC16,TNFSF10
Non-germinomatous germ cell tumor	2.46	0.0847	1/59 (2%)	0/59 (0%)	4/59 (7%)	54/59 (92%)	HSP90AA1,IRF1,KLF4,MUC16,NBN
Graft-vs-host disease	2.44	0.108	0/37 (0%)	0/37 (0%)	4/37 (11%)	33/37 (89%)	BCL6,IL1A,IL1RL1,NFE2L2
Infection by herpes simplex virus	2.44	0.108	0/37 (0%)	0/37 (0%)	4/37 (11%)	33/37 (89%)	IL15,IRF1,SAMHD1,TAP1
Hemosiderosis	2.44	0.108	1/37 (3%)	0/37 (0%)	3/37 (8%)	33/37 (89%)	CEBPA,CXCL10,IL18BP,SMN1/SMN2
Listeriosis	2.44	0.108	0/37 (0%)	0/37 (0%)	4/37 (11%)	33/37 (89%)	IL6,IRF9,NLRC5,SOCS1
Lymphocytic neoplasm	2.44	0.108	0/37 (0%)	0/37 (0%)	4/37 (11%)	33/37 (89%)	CCL2,CYP1A1,SERPINB2,TNFSF10
Dystonia	2.4	0.158	0/19 (0%)	0/19 (0%)	3/19 (16%)	16/19 (84%)	GCH1,NOCT,THAP1
Pulmonary Hypertension	2.39	0.105	0/38 (0%)	0/38 (0%)	4/38 (11%)	34/38 (89%)	EDNRA,IL15,STAT1,TNFSF10
Contact dermatitis	2.39	0.105	0/38 (0%)	0/38 (0%)	4/38 (11%)	34/38 (89%)	ANXA1,CIITA,NLRC5,PARP9
Fibroma	2.39	0.105	0/38 (0%)	0/38 (0%)	4/38 (11%)	34/38 (89%)	CTNNAL1,HMMR,HSPE1,OAS1
Chronic thrombocytosis	2.39	0.105	0/38 (0%)	0/38 (0%)	4/38 (11%)	34/38 (89%)	IRF1,JAK2,NLRC5,STAT1
Fatal infantile encephalopathy or pulmonary hypertension	2.39	0.105	0/38 (0%)	0/38 (0%)	4/38 (11%)	34/38 (89%)	EDNRA,IL15,STAT1,TNFSF10
Teratoma	2.37	0.0806	1/62 (2%)	0/62 (0%)	4/62 (6%)	57/62 (92%)	HSP90AA1,IRF1,KLF4,MUC16,NBN
Malignant solid organ tumor	2.37	0.0806	0/62 (0%)	0/62 (0%)	5/62 (8%)	57/62 (92%)	EPSTI1,FERMT2,MLF1,NMI,WARS1
Mature B cell malignant tumor	2.37	0.0806	0/62 (0%)	0/62 (0%)	5/62 (8%)	57/62 (92%)	BCL6,HSP90AA1,IL6,KLF4,SNRPE
Colitis	2.35	0.103	0/39 (0%)	0/39 (0%)	4/39 (10%)	35/39 (90%)	CD274,HMOX1,JAK2,RIGI
Rheumatic disease of joint	2.35	0.103	0/39 (0%)	0/39 (0%)	4/39 (10%)	35/39 (90%)	CASP1,IL1A,IL6,RIPK2
Recurrent malignant solid tumor	2.35	0.103	0/39 (0%)	0/39 (0%)	4/39 (10%)	35/39 (90%)	BCL6,IRF1,NLRC5,STAT1
Severe connective tissue disorder	2.35	0.103	0/39 (0%)	0/39 (0%)	4/39 (10%)	35/39 (90%)	BCL6,FAS,HSPE1,JAK2
Non-traumatic arthropathy	2.35	0.103	0/39 (0%)	0/39 (0%)	4/39 (10%)	35/39 (90%)	IL15,IL1A,IL6,TNFRSF1B
Mature B-cell lymphoma	2.34	0.0794	0/63 (0%)	0/63 (0%)	5/63 (8%)	58/63 (92%)	BCL6,HSP90AA1,KLF4,NOC3L,PMAIP1

Acute pancreatitis	2.31	0.1	0/40 (0%)	0/40 (0%)	4/40 (10%)	36/40 (90%)	ATG5,IFT57,STAT1,UGDH
Neuroinflammation	2.31	0.1	0/40 (0%)	0/40 (0%)	4/40 (10%)	36/40 (90%)	GBP5,IL1A,IL6,NBN
Osteoporosis	2.27	0.0976	1/41 (2%)	0/41 (0%)	3/41 (7%)	37/41 (90%)	B2M,BCL6,BGN,IRF9
Erythema	2.27	0.0976	1/41 (2%)	0/41 (0%)	3/41 (7%)	37/41 (90%)	HIF1A,HSPE1,NOXO1,SLC2A3
Toxoplasmosis	2.27	0.0976	0/41 (0%)	0/41 (0%)	4/41 (10%)	37/41 (90%)	GBP2,PNPT1,SOCS1,TNFSF10
Fungal Infection	2.27	0.0976	0/41 (0%)	0/41 (0%)	4/41 (10%)	37/41 (90%)	IL1A,IL6,STAT1,TNFRSF1B
Gastrointestinal tract cancer	2.27	0.0976	1/41 (2%)	0/41 (0%)	3/41 (7%)	37/41 (90%)	NBN,PRODH,STAT1,TNFSF10
Low bone density	2.27	0.0976	1/41 (2%)	0/41 (0%)	3/41 (7%)	37/41 (90%)	B2M,BCL6,BGN,IRF9
Cerebrovascular dysfunction	2.24	0.0952	2/42 (5%)	0/42 (0%)	2/42 (5%)	38/42 (90%)	HIF1A,NOTCH3,PDCD10,VWF
Salmonellosis	2.24	0.0952	0/42 (0%)	0/42 (0%)	4/42 (10%)	38/42 (90%)	CASP1,IL15,STAT1,TNFRSF1B
Methylmalonic acidemia	2.24	0.0952	1/42 (2%)	0/42 (0%)	3/42 (7%)	38/42 (90%)	HSPE1,HSPH1,MMAA,SSTR5
Ophthalmia	2.24	0.0952	0/42 (0%)	0/42 (0%)	4/42 (10%)	38/42 (90%)	HAT1,PRDX3,STAT1,TNFRSF1B
Enteritis	2.21	0.136	0/22 (0%)	0/22 (0%)	3/22 (14%)	19/22 (86%)	CD274,HMOX1,JAK2
Progression of tumor	2.2	0.093	0/43 (0%)	0/43 (0%)	4/43 (9%)	39/43 (91%)	MLF1,NMI,SP100,TNFSF10
Prostatic intraepithelial tumor	2.2	0.093	0/43 (0%)	0/43 (0%)	4/43 (9%)	39/43 (91%)	CUL2,NKX3-1,NMI,SOCS3
Pneumonia	2.2	0.093	0/43 (0%)	0/43 (0%)	4/43 (9%)	39/43 (91%)	GBP5,HIF1A,IL6,STAT1
Septicemia	2.2	0.093	0/43 (0%)	0/43 (0%)	4/43 (9%)	39/43 (91%)	C4A/C4B,CASP1,ICAM1,STAT1
Cardiovascular neoplasm	2.2	0.093	1/43 (2%)	0/43 (0%)	3/43 (7%)	39/43 (91%)	FGF1,IL1A,IL6,PDCD10
Cholangiocarcinoma	2.2	0.093	1/43 (2%)	0/43 (0%)	3/43 (7%)	39/43 (91%)	CD274,IL6,NOTCH3,TNFSF10
Biliary tract tumor	2.2	0.093	1/43 (2%)	0/43 (0%)	3/43 (7%)	39/43 (91%)	CD274,IL6,NOTCH3,TNFSF10
Biliary tract carcinoma	2.2	0.093	1/43 (2%)	0/43 (0%)	3/43 (7%)	39/43 (91%)	CD274,IL6,NOTCH3,TNFSF10
Chronic inflammatory arthropathy	2.17	0.0909	0/44 (0%)	0/44 (0%)	4/44 (9%)	40/44 (91%)	CIITA,IL15,IL1A,IL6
Bile duct carcinoma	2.17	0.0909	1/44 (2%)	0/44 (0%)	3/44 (7%)	40/44 (91%)	CD274,IL6,NOTCH3,TNFSF10
Uterine carcinoma	2.17	0.0909	1/44 (2%)	0/44 (0%)	3/44 (7%)	40/44 (91%)	CEBPA,HAT1,KLF4,TOP1
Histiocytosis	2.17	0.0909	0/44 (0%)	0/44 (0%)	4/44 (9%)	40/44 (91%)	IL1A,IL6,NMI,STX11
Bile duct cancer	2.17	0.0909	1/44 (2%)	0/44 (0%)	3/44 (7%)	40/44 (91%)	CD274,IL6,NOTCH3,TNFSF10
Bile duct tumor	2.17	0.0909	1/44 (2%)	0/44 (0%)	3/44 (7%)	40/44 (91%)	CD274,IL6,NOTCH3,TNFSF10
Chronic arthropathy	2.17	0.0909	0/44 (0%)	0/44 (0%)	4/44 (9%)	40/44 (91%)	CIITA,IL15,IL1A,IL6
Bile duct adenocarcinoma	2.17	0.0909	1/44 (2%)	0/44 (0%)	3/44 (7%)	40/44 (91%)	CD274,IL6,NOTCH3,TNFSF10
Biliary tract adenocarcinoma	2.17	0.0909	1/44 (2%)	0/44 (0%)	3/44 (7%)	40/44 (91%)	CD274,IL6,NOTCH3,TNFSF10
Restenosis	2.16	0.13	0/23 (0%)	0/23 (0%)	3/23 (13%)	20/23 (87%)	CCL2,HSPE1,SERPINB2
Progression of glioma	2.13	0.0889	0/45 (0%)	0/45 (0%)	4/45 (9%)	41/45 (91%)	HAT1,HIF1A,IL6,SERPINB2
Progression of neuroepithelial tumor	2.13	0.0889	0/45 (0%)	0/45 (0%)	4/45 (9%)	41/45 (91%)	HAT1,HIF1A,IL6,SERPINB2
Glandular intraepithelial neoplasm	2.13	0.0889	0/45 (0%)	0/45 (0%)	4/45 (9%)	41/45 (91%)	NAE1,NBN,NKX3-1,SOCS3
Progressive central nervous system tumor	2.13	0.0889	0/45 (0%)	0/45 (0%)	4/45 (9%)	41/45 (91%)	HAT1,HIF1A,IL6,SERPINB2
Development of ovarian tumor	2.1	0.087	0/46 (0%)	0/46 (0%)	4/46 (9%)	42/46 (91%)	B2M,CD274,FAS,ME1
Combined pituitary hormone deficiency	2.1	0.087	0/46 (0%)	0/46 (0%)	4/46 (9%)	42/46 (91%)	EIF2S2,HSPE1,NBN,TPT1
Male genital neoplasm	2.1	0.087	0/46 (0%)	0/46 (0%)	4/46 (9%)	42/46 (91%)	ATF3,HSPE1,MLF1,NBN
Subcutaneous tumor	2.1	0.087	0/46 (0%)	0/46 (0%)	4/46 (9%)	42/46 (91%)	GEMIN2,HSPA6,NMI,SMN1/SMN2
BCR-ABL negative myeloid neoplasm	2.06	0.0851	0/47 (0%)	0/47 (0%)	4/47 (9%)	43/47 (91%)	IRF1,JAK2,NLRC5,STAT1
Philadelphia chromosome negative hematological system tumor	2.06	0.0851	0/47 (0%)	0/47 (0%)	4/47 (9%)	43/47 (91%)	IRF1,JAK2,NLRC5,STAT1
Philadelphia chromosome negative chronic myeloproliferative neoplasm	2.06	0.0851	0/47 (0%)	0/47 (0%)	4/47 (9%)	43/47 (91%)	IRF1,JAK2,NLRC5,STAT1
Combined pituitary hormone deficiency or pituitary stalk interruption syndrome	2.06	0.0851	0/47 (0%)	0/47 (0%)	4/47 (9%)	43/47 (91%)	EIF2S2,HSPE1,NBN,TPT1
Oral cancer	2.03	0.0833	1/48 (2%)	0/48 (0%)	3/48 (6%)	44/48 (92%)	CD274,HAT1,HSP90AA1,PRODH
Thymoma	2.01	0.115	0/26 (0%)	0/26 (0%)	3/26 (12%)	23/26 (88%)	CASP1,HSPH1,XAF1
Arrhythmia of heart ventricle	2.01	0.115	0/26 (0%)	0/26 (0%)	3/26 (12%)	23/26 (88%)	APOL6,HSPE1,SNRPE
Inflammatory arthropathy	2	0.0816	0/49 (0%)	0/49 (0%)	4/49 (8%)	45/49 (92%)	CASP1,DUSP1,IL1A,IL6
Arthralgia/arthritis	2	0.0816	0/49 (0%)	0/49 (0%)	4/49 (8%)	45/49 (92%)	CASP1,DUSP1,IL1A,IL6
Primitive neuroectodermal brain tumor	2	0.0816	0/49 (0%)	0/49 (0%)	4/49 (8%)	45/49 (92%)	FERMT2,HSP90AA1,RAB23,SLFN11
B-cell chronic lymphoproliferative disorder	2	0.0816	0/49 (0%)	0/49 (0%)	4/49 (8%)	45/49 (92%)	IRF1,KLF4,STAT1,TNFSF10
Chronic lymphoproliferative disease	2	0.0816	0/49 (0%)	0/49 (0%)	4/49 (8%)	45/49 (92%)	IRF1,KLF4,STAT1,TNFSF10
B-cell neoplasm	2	0.0816	0/49 (0%)	0/49 (0%)	4/49 (8%)	45/49 (92%)	HSP90AA1,IL6,NBN,NMI
Central nervous system primitive neuroectodermal tumor	2	0.0816	0/49 (0%)	0/49 (0%)	4/49 (8%)	45/49 (92%)	FERMT2,HSP90AA1,RAB23,SLFN11
Intracranial embryonal tumor	1.97	0.08	0/50 (0%)	0/50 (0%)	4/50 (8%)	46/50 (92%)	FERMT2,HSP90AA1,RAB23,SLFN11
Endotoxemia	1.97	0.08	0/50 (0%)	0/50 (0%)	4/50 (8%)	46/50 (92%)	C4A/C4B,CASP1,ICAM1,STAT1
Endotoxicosis	1.97	0.08	0/50 (0%)	0/50 (0%)	4/50 (8%)	46/50 (92%)	C4A/C4B,CASP1,ICAM1,STAT1
Embryonal tumor of brain	1.97	0.08	0/50 (0%)	0/50 (0%)	4/50 (8%)	46/50 (92%)	FERMT2,HSP90AA1,RAB23,SLFN11
Ulceration of skin	1.96	0.111	0/27 (0%)	0/27 (0%)	3/27 (11%)	24/27 (89%)	CASP1,CXCL10,IL1RL1
Ventricular tachycardia	1.96	0.111	0/27 (0%)	0/27 (0%)	3/27 (11%)	24/27 (89%)	APOL6,HSPE1,SNRPE
Gastrointestinal ulcer	1.96	0.111	0/27 (0%)	0/27 (0%)	3/27 (11%)	24/27 (89%)	ANXA1,CUL2,SERPINB2
Humoral immunodeficiency	1.96	0.111	0/27 (0%)	0/27 (0%)	3/27 (11%)	24/27 (89%)	BCL6,CIITA,HSPH1

Infection by Citrobacter	1.96	0.111	0/27 (0%)	0/27 (0%)	3/27 (11%)	24/27 (89%)	EPSTI1,IL6,STAT1
Thyroid gland tumor	1.94	0.0784	0/51 (0%)	0/51 (0%)	4/51 (8%)	47/51 (92%)	HSPE1,NBN,SERPINB2,SNRPE
Gastrointestinal adenocarcinoma	1.94	0.0784	1/51 (2%)	0/51 (0%)	3/51 (6%)	47/51 (92%)	B2M,DNAJA4,NBN,PARD6A
Prostatic tumor	1.94	0.0784	0/51 (0%)	0/51 (0%)	4/51 (8%)	47/51 (92%)	ATF3,MLF1,NBN,NKX3-1
Dryness of skin	1.92	0.107	1/28 (4%)	0/28 (0%)	2/28 (7%)	25/28 (89%)	GBP5,RIPK2,SSTR5
Female genital neoplasm	1.91	0.0769	1/52 (2%)	0/52 (0%)	3/52 (6%)	48/52 (92%)	CEBPA,CYP1A1,CYP1B1,SLFN11
Inborn error of amino acid metabolism	1.88	0.0755	0/53 (0%)	0/53 (0%)	4/53 (8%)	49/53 (92%)	SERPINB2,SMN1/SMN2,TIA1,XAF1
Plasma cell neoplasm	1.88	0.0755	0/53 (0%)	0/53 (0%)	4/53 (8%)	49/53 (92%)	HSP90AA1,IL6,KLF4,SNRPE
Aggressive lymphoma	1.88	0.0755	1/53 (2%)	0/53 (0%)	3/53 (6%)	49/53 (92%)	BCL6,KLF4,NOC3L,RIPK4
Occlusion of carotid artery	1.88	0.103	0/29 (0%)	0/29 (0%)	3/29 (10%)	26/29 (90%)	FERMT2,HSPE1,PDCD10
Combined immunodeficiency	1.88	0.103	0/29 (0%)	0/29 (0%)	3/29 (10%)	26/29 (90%)	BCL6,CIITA,HSPH1
Severe combined immunodeficiency	1.88	0.103	0/29 (0%)	0/29 (0%)	3/29 (10%)	26/29 (90%)	BCL6,CIITA,HSPH1
Severe immunodeficiency	1.88	0.103	0/29 (0%)	0/29 (0%)	3/29 (10%)	26/29 (90%)	BCL6,CIITA,HSPH1
Severe primary immunodeficiency disorder	1.88	0.103	0/29 (0%)	0/29 (0%)	3/29 (10%)	26/29 (90%)	BCL6,CIITA,HSPH1
Deficiency of cell-mediated immunity	1.88	0.103	0/29 (0%)	0/29 (0%)	3/29 (10%)	26/29 (90%)	BCL6,CIITA,HSPH1
Mastocytoma	1.86	0.0741	1/54 (2%)	0/54 (0%)	3/54 (6%)	50/54 (93%)	FAS,HSP90AA1,NOTCH3,TNFRSF1B
Mature B-cell neoplasm	1.86	0.0741	0/54 (0%)	0/54 (0%)	4/54 (7%)	50/54 (93%)	BCL6,HSP90AA1,IL6,SNRPE
Multi-organ cancers	1.86	0.0741	1/54 (2%)	0/54 (0%)	3/54 (6%)	50/54 (93%)	IRF1,MUC16,SLFN11,STAT1
Abdominal cyst	1.84	0.1	0/30 (0%)	0/30 (0%)	3/30 (10%)	27/30 (90%)	HIF1A,KLF4,RSAD2
Basal-cell tumor	1.83	0.0727	0/55 (0%)	0/55 (0%)	4/55 (7%)	51/55 (93%)	FERMT2,IFT57,IL6,MLF1
Pancytopenia	1.83	0.0727	1/55 (2%)	0/55 (0%)	3/55 (5%)	51/55 (93%)	GBP5,NBN,SAMD9L,VWF
Cystic kidney disease	1.83	0.0727	1/55 (2%)	0/55 (0%)	3/55 (5%)	51/55 (93%)	HIF1A,SOCS1,SSTR5,XAF1
Breast adenocarcinoma	1.83	0.0727	0/55 (0%)	0/55 (0%)	4/55 (7%)	51/55 (93%)	CUL2,HSP90AA1,IRF1,STAT1
Nondiabetic nephropathy	1.83	0.0727	1/55 (2%)	0/55 (0%)	3/55 (5%)	51/55 (93%)	HIF1A,SOCS1,SSTR5,XAF1
Eye neoplasm	1.8	0.0714	0/56 (0%)	0/56 (0%)	4/56 (7%)	52/56 (93%)	HSP90AA1,MLF1,SNRPE,TOP1
Adrenal gland tumor	1.78	0.0702	1/57 (2%)	0/57 (0%)	3/57 (5%)	53/57 (93%)	CEBPA,FERMT2,HAT1,KLF4
Periadrenal tumor	1.78	0.0702	1/57 (2%)	0/57 (0%)	3/57 (5%)	53/57 (93%)	CEBPA,FERMT2,HAT1,KLF4
Sun-shielded melanoma	1.78	0.0702	1/57 (2%)	0/57 (0%)	3/57 (5%)	53/57 (93%)	IRF1,NLRC5,NOTCH3,STAT1
Acute respiratory distress syndrome	1.76	0.0938	0/32 (0%)	0/32 (0%)	3/32 (9%)	29/32 (91%)	IL6,JAK2,NFE2L2
Purpura	1.76	0.0938	1/32 (3%)	0/32 (0%)	2/32 (6%)	29/32 (91%)	C4A/C4B,SOCS1,VWF
High grade lymphoid cancer	1.75	0.069	1/58 (2%)	0/58 (0%)	3/58 (5%)	54/58 (93%)	BCL6,KLF4,NOC3L,RIPK4
High grade lymphocytic cancer	1.75	0.069	1/58 (2%)	0/58 (0%)	3/58 (5%)	54/58 (93%)	BCL6,KLF4,NOC3L,RIPK4
Aggressive B cell non-Hodgkin lymphoma	1.75	0.069	1/58 (2%)	0/58 (0%)	3/58 (5%)	54/58 (93%)	BCL6,KLF4,NOC3L,RIPK4
Aggressive non-Hodgkin lymphoma	1.75	0.069	1/58 (2%)	0/58 (0%)	3/58 (5%)	54/58 (93%)	BCL6,KLF4,NOC3L,RIPK4
Aggressive mature B-cell lymphoma	1.75	0.069	1/58 (2%)	0/58 (0%)	3/58 (5%)	54/58 (93%)	BCL6,KLF4,NOC3L,RIPK4
Aggressive B-cell lymphoma	1.75	0.069	1/58 (2%)	0/58 (0%)	3/58 (5%)	54/58 (93%)	BCL6,KLF4,NOC3L,RIPK4
Large-cell lymphoma	1.73	0.0678	0/59 (0%)	0/59 (0%)	4/59 (7%)	55/59 (93%)	BCL6,KLF4,NOC3L,PMAIP1
Mixed neoplasia	1.73	0.0678	1/59 (2%)	0/59 (0%)	3/59 (5%)	55/59 (93%)	CEBPA,KLF4,NMI,SLC2A3
Hypoproteinemia	1.72	0.0909	0/33 (0%)	0/33 (0%)	3/33 (9%)	30/33 (91%)	B2M,C4A/C4B,PMAIP1
Steatorrhea	1.72	0.0909	1/33 (3%)	0/33 (0%)	2/33 (6%)	30/33 (91%)	ATF3,DUSP9,IL1A
Reperfusion injury	1.72	0.0909	0/33 (0%)	0/33 (0%)	3/33 (9%)	30/33 (91%)	ANXA1,CD55,RIPK2
Development of primitive neuroectodermal tumor	1.7	0.0667	0/60 (0%)	0/60 (0%)	4/60 (7%)	56/60 (93%)	FERMT2,NBN,TNFSF10,TOP1
Development of neuroectodermal tumor	1.7	0.0667	0/60 (0%)	0/60 (0%)	4/60 (7%)	56/60 (93%)	FERMT2,NBN,TNFSF10,TOP1
Angioedema	1.69	0.0882	0/34 (0%)	0/34 (0%)	3/34 (9%)	31/34 (91%)	C1R,C1S,SERPING1
Enterocolitis	1.69	0.0882	0/34 (0%)	0/34 (0%)	3/34 (9%)	31/34 (91%)	DUSP1,MLF1,TAP1
Herpes simplex encephalitis	1.69	0.0882	0/34 (0%)	0/34 (0%)	3/34 (9%)	31/34 (91%)	IRF1,LYN,STAT1
Non-Hodgkin lymphoma	1.68	0.0656	0/61 (0%)	0/61 (0%)	4/61 (7%)	57/61 (93%)	BCL6,MLF1,NBN,SLFN11
Ulcerative or indeterminate colitis	1.66	0.0857	0/35 (0%)	0/35 (0%)	3/35 (9%)	32/35 (91%)	BCL6,CASP1,JAK2
Colon carcinoma	1.66	0.0857	0/35 (0%)	0/35 (0%)	3/35 (9%)	32/35 (91%)	CD274,IL1A,TOP1
Erythrocytosis	1.66	0.0857	0/35 (0%)	0/35 (0%)	3/35 (9%)	32/35 (91%)	HIF1A,JAK2,SOCS3
Ulcerative colitis	1.66	0.0857	0/35 (0%)	0/35 (0%)	3/35 (9%)	32/35 (91%)	BCL6,CASP1,JAK2
Acute renal failure	1.66	0.0857	0/35 (0%)	0/35 (0%)	3/35 (9%)	32/35 (91%)	CD274,HMOX1,ZFP36
Acute renal insufficiency	1.66	0.0857	0/35 (0%)	0/35 (0%)	3/35 (9%)	32/35 (91%)	CD274,HMOX1,ZFP36
Hereditary skeletal myopathy	1.62	0.0833	2/36 (6%)	0/36 (0%)	1/36 (3%)	33/36 (92%)	BGN,MGAT3,SMN1/SMN2
Lymphoid cancer	1.62	0.0833	0/36 (0%)	0/36 (0%)	3/36 (8%)	33/36 (92%)	CCL2,CYP1A1,TNFSF10
Immunodeficiency	1.62	0.0833	0/36 (0%)	0/36 (0%)	3/36 (8%)	33/36 (92%)	CIITA,IL15,NBN
Systemic inflammatory response syndrome	1.62	0.0833	1/36 (3%)	0/36 (0%)	2/36 (6%)	33/36 (92%)	ANXA1,CASP1,VWF
Malignant solid tumor	1.62	0.0833	0/36 (0%)	0/36 (0%)	3/36 (8%)	33/36 (92%)	FERMT2,NMI,TNFSF10
Coagulation factor deficiency syndrome	1.62	0.0833	1/36 (3%)	0/36 (0%)	2/36 (6%)	33/36 (92%)	BATF2,PRDM1,VWF
Hepatotoxicity	1.59	0.0811	0/37 (0%)	0/37 (0%)	3/37 (8%)	34/37 (92%)	IL15,NFE2L2,TNFRSF1B
Non-melanoma solid tumor	1.59	0.0811	0/37 (0%)	0/37 (0%)	3/37 (8%)	34/37 (92%)	FERMT2,MLF1,NMI

Malignant genitourinary solid tumor	1.59	0.0811	2/37 (5%)	0/37 (0%)	1/37 (3%)	34/37 (92%)	CEBPA,MUC16,STAT1
Systemic lupus erythematosus	1.56	0.0789	0/38 (0%)	0/38 (0%)	3/38 (8%)	35/38 (92%)	IL15,IL6,LYN
Infection by respiratory syncytial virus	1.56	0.0789	0/38 (0%)	0/38 (0%)	3/38 (8%)	35/38 (92%)	CCL2,IFIT2,IL1A
Carditis	1.56	0.0789	0/38 (0%)	0/38 (0%)	3/38 (8%)	35/38 (92%)	BCL6,IL1A,STAT1
Relapsed neoplasia	1.56	0.0789	0/38 (0%)	0/38 (0%)	3/38 (8%)	35/38 (92%)	IRF1,NLRC5,STAT1
Pancreaticobiliary neoplasm	1.56	0.0789	0/38 (0%)	0/38 (0%)	3/38 (8%)	35/38 (92%)	HIF1A,IL6,TNFSF10
Pancreatobiliary tumor	1.56	0.0789	0/38 (0%)	0/38 (0%)	3/38 (8%)	35/38 (92%)	HIF1A,IL6,TNFSF10
Supra-aortic artery disease	1.56	0.0789	0/38 (0%)	0/38 (0%)	3/38 (8%)	35/38 (92%)	FERMT2,HSPE1,SMN1/SMN2
Synovitis	1.53	0.0769	0/39 (0%)	0/39 (0%)	3/39 (8%)	36/39 (92%)	CASP1,IL1A,IRF1
Mast cell neoplasm	1.53	0.0769	0/39 (0%)	0/39 (0%)	3/39 (8%)	36/39 (92%)	CASP1,HSP90AA1,HSPH1
Parasitic Infection	1.53	0.0769	0/39 (0%)	0/39 (0%)	3/39 (8%)	36/39 (92%)	IL15,IL1A,IL6
Immediate hypersensitivity	1.53	0.0769	1/39 (3%)	0/39 (0%)	2/39 (5%)	36/39 (92%)	FLG,IL6,PDCD1LG2
Atopy	1.53	0.0769	1/39 (3%)	0/39 (0%)	2/39 (5%)	36/39 (92%)	FLG,IL6,PDCD1LG2
Recurrent neoplasm	1.53	0.0769	0/39 (0%)	0/39 (0%)	3/39 (8%)	36/39 (92%)	IRF1,NLRC5,STAT1
Recurrent solid tumor	1.53	0.0769	0/39 (0%)	0/39 (0%)	3/39 (8%)	36/39 (92%)	IRF1,NLRC5,STAT1
Polydipsia	1.52	0.125	1/16 (6%)	0/16 (0%)	1/16 (6%)	14/16 (88%)	PRDX3,RIPK4
Bullous dermatitis	1.5	0.075	0/40 (0%)	0/40 (0%)	3/40 (8%)	37/40 (93%)	C4A/C4B,HAT1,IL1RL1
Ovarian adenocarcinoma	1.5	0.075	0/40 (0%)	0/40 (0%)	3/40 (8%)	37/40 (93%)	B2M,CD274,FAS
Autoimmune bullous skin disease	1.48	0.0732	0/41 (0%)	0/41 (0%)	3/41 (7%)	38/41 (93%)	C4A/C4B,HAT1,IL1RL1
Cachexia	1.48	0.0732	0/41 (0%)	0/41 (0%)	3/41 (7%)	38/41 (93%)	HMOX1,IL6,TNFRSF1B
Deterioration of connective tissue	1.48	0.0732	0/41 (0%)	0/41 (0%)	3/41 (7%)	38/41 (93%)	IL1A,IL6,NAMPT
Progressive motor neuropathy	1.48	0.0732	0/41 (0%)	0/41 (0%)	3/41 (7%)	38/41 (93%)	GCH1,SAMHD1,WARS1
Infection by mycobacteria	1.48	0.0732	0/41 (0%)	0/41 (0%)	3/41 (7%)	38/41 (93%)	IL15,IL1A,STAT1
Progressive motor neuron disease	1.48	0.0732	0/41 (0%)	0/41 (0%)	3/41 (7%)	38/41 (93%)	GCH1,SAMHD1,WARS1
Ovarian lesion	1.48	0.0732	0/41 (0%)	0/41 (0%)	3/41 (7%)	38/41 (93%)	CYP1A1,DUSP1,TIA1
Progressive neuropathy	1.48	0.0732	0/41 (0%)	0/41 (0%)	3/41 (7%)	38/41 (93%)	GCH1,SAMHD1,WARS1
Hepato-pancreato-biliary cancer	1.45	0.0714	0/42 (0%)	0/42 (0%)	3/42 (7%)	39/42 (93%)	IL6,NMI,TNFSF10
Ichthyosis	1.45	0.0714	2/42 (5%)	0/42 (0%)	1/42 (2%)	39/42 (93%)	RIPK2,TGM1,TINCR
X-linked hereditary disease	1.45	0.0714	1/42 (2%)	0/42 (0%)	2/42 (5%)	39/42 (93%)	NBN,PRDM1,VWF
Sex-Linked Hereditary Disorder	1.45	0.0714	1/42 (2%)	0/42 (0%)	2/42 (5%)	39/42 (93%)	NBN,PRDM1,VWF
Retinal neovascularization	1.45	0.0714	0/42 (0%)	0/42 (0%)	3/42 (7%)	39/42 (93%)	SOCS3,WARS1,ZFP36
Liver carcinoma	1.45	0.0714	0/42 (0%)	0/42 (0%)	3/42 (7%)	39/42 (93%)	IL1A,IL6,NMI
Disrupted blood brain barrier	1.45	0.0714	0/42 (0%)	0/42 (0%)	3/42 (7%)	39/42 (93%)	IL1A,IL6,STAT1
Tuberculosis	1.42	0.0698	0/43 (0%)	0/43 (0%)	3/43 (7%)	40/43 (93%)	IL15,IL1A,TICAM2
Conjunctivitis	1.42	0.0698	0/43 (0%)	0/43 (0%)	3/43 (7%)	40/43 (93%)	HAT1,ICAM1,ZFP36
Sarcoma	1.42	0.0698	0/43 (0%)	0/43 (0%)	3/43 (7%)	40/43 (93%)	ATF3,IL6,NMI
Hyperkeratosis	1.42	0.0698	1/43 (2%)	0/43 (0%)	2/43 (5%)	40/43 (93%)	HAT1,IL1A,TINCR
Respiratory failure	1.42	0.0698	1/43 (2%)	0/43 (0%)	2/43 (5%)	40/43 (93%)	C4A/C4B,HAT1,PRRX2
Renal lesion	1.42	0.0698	0/43 (0%)	0/43 (0%)	3/43 (7%)	40/43 (93%)	ACE2,CYP1A1,HIF1A
Head and neck squamous cell carcinoma	1.42	0.0698	0/43 (0%)	0/43 (0%)	3/43 (7%)	40/43 (93%)	CD274,HSP90AA1,TICAM2
Central nervous system hemangioma	1.42	0.0698	0/43 (0%)	0/43 (0%)	3/43 (7%)	40/43 (93%)	ICAM1,KLF4,PDCD10
Head and neck squamous tumor	1.42	0.0698	0/43 (0%)	0/43 (0%)	3/43 (7%)	40/43 (93%)	CD274,HSP90AA1,TICAM2
Malignant connective or soft tissue neoplasm	1.42	0.0698	0/43 (0%)	0/43 (0%)	3/43 (7%)	40/43 (93%)	IL6,IRF1,NMI
Enterobacteriaceae infection	1.42	0.0698	0/43 (0%)	0/43 (0%)	3/43 (7%)	40/43 (93%)	CASP1,IL15,STAT1
Esophagus tumor	1.4	0.0682	1/44 (2%)	0/44 (0%)	2/44 (5%)	41/44 (93%)	KCNN4,NFE2L2,TXNRD1
Ischemia of brain	1.4	0.0682	1/44 (2%)	0/44 (0%)	2/44 (5%)	41/44 (93%)	HIF1A,HSPH1,VWF
Hepatoblastoma	1.4	0.0682	0/44 (0%)	0/44 (0%)	3/44 (7%)	41/44 (93%)	HSP90AA1,KLF4,NBN
Cirrhosis of liver	1.4	0.0682	1/44 (2%)	0/44 (0%)	2/44 (5%)	41/44 (93%)	EIF2S2,SREBF1,STAT1
Cirrhosis	1.4	0.0682	1/44 (2%)	0/44 (0%)	2/44 (5%)	41/44 (93%)	EIF2S2,SREBF1,STAT1
Formation of renal lesion	1.4	0.0682	0/44 (0%)	0/44 (0%)	3/44 (7%)	41/44 (93%)	ATF3,HIF1A,SLC2A3
Colon adenocarcinoma	1.4	0.0682	0/44 (0%)	0/44 (0%)	3/44 (7%)	41/44 (93%)	CXCL10,NBN,TOP1
Ventricular dilatation	1.4	0.0682	0/44 (0%)	0/44 (0%)	3/44 (7%)	41/44 (93%)	IRF1,RIGI,STAT1
Ductal carcinoma	1.4	0.0682	0/44 (0%)	0/44 (0%)	3/44 (7%)	41/44 (93%)	CD274,IL6,TNFSF10
Gastroesophageal adenocarcinoma	1.4	0.0682	0/44 (0%)	0/44 (0%)	3/44 (7%)	41/44 (93%)	CD274,HSP90AA1,RIPK2
Advanced fibrosis	1.4	0.0682	1/44 (2%)	0/44 (0%)	2/44 (5%)	41/44 (93%)	EIF2S2,SREBF1,STAT1
Advanced hepatic fibrosis	1.4	0.0682	1/44 (2%)	0/44 (0%)	2/44 (5%)	41/44 (93%)	EIF2S2,SREBF1,STAT1
Endometrial carcinoma	1.37	0.0667	1/45 (2%)	0/45 (0%)	2/45 (4%)	42/45 (93%)	CEBPA,HAT1,KLF4
Polyarthritis	1.37	0.0667	0/45 (0%)	0/45 (0%)	3/45 (7%)	42/45 (93%)	DUSP1,IL1A,IL6
Hyperphenylalaninemia	1.37	0.0667	0/45 (0%)	0/45 (0%)	3/45 (7%)	42/45 (93%)	GCH1,NOCT,SMN1/SMN2
Candidiasis	1.37	0.0667	1/45 (2%)	0/45 (0%)	2/45 (4%)	42/45 (93%)	GBP5,KCNN4,STAT1
Venoocclusion	1.37	0.0667	0/45 (0%)	0/45 (0%)	3/45 (7%)	42/45 (93%)	HSPE1,SP100,SP110

Anterior segment inflammation	1.37	0.0667	0/45 (0%)	0/45 (0%)	3/45 (7%)	42/45 (93%)	ANXA1,CDKN3,SERPINB2
Pancreaticobiliary cancer	1.37	0.0667	1/45 (2%)	0/45 (0%)	2/45 (4%)	42/45 (93%)	IL6,TNFSF10,VWF
Occurrence of tumor	1.35	0.0652	0/46 (0%)	0/46 (0%)	3/46 (7%)	43/46 (93%)	CASP1,IL1A,SERPINB2
Formation of uterine carcinoma	1.35	0.0652	1/46 (2%)	0/46 (0%)	2/46 (4%)	43/46 (93%)	CEBPA,HAT1,KLF4
Vasculitis	1.35	0.0652	0/46 (0%)	0/46 (0%)	3/46 (7%)	43/46 (93%)	HSPE1,JAK2,ZFP36
Infection by Influenza virus	1.35	0.0652	0/46 (0%)	0/46 (0%)	3/46 (7%)	43/46 (93%)	IL6,IRF1,STAT1
Nerve sheath tumor	1.35	0.0652	1/46 (2%)	0/46 (0%)	2/46 (4%)	43/46 (93%)	HSP90AA1,MLF1,MUC16
Advanced melanoma	1.35	0.0652	0/46 (0%)	0/46 (0%)	3/46 (7%)	43/46 (93%)	CUL2,MLF1,TIA1
Idiopathic pulmonary fibrosis	1.34	0.1	0/20 (0%)	0/20 (0%)	2/20 (10%)	18/20 (90%)	EDNRA,HSPH1
Female genital carcinoma	1.33	0.0638	1/47 (2%)	0/47 (0%)	2/47 (4%)	44/47 (94%)	CEBPA,FAS,KLF4
B-cell lymphoproliferative disorder	1.33	0.0638	0/47 (0%)	0/47 (0%)	3/47 (6%)	44/47 (94%)	HSP90AA1,IL6,NBN
Development of endocrine gland tumor	1.33	0.0638	1/47 (2%)	0/47 (0%)	2/47 (4%)	44/47 (94%)	CEBPA,OAS1,SNRPE
Diabetes mellitus	1.33	0.0638	1/47 (2%)	0/47 (0%)	2/47 (4%)	44/47 (94%)	CD274,SOCS1,ZBED6
Keratosis	1.33	0.0638	1/47 (2%)	0/47 (0%)	2/47 (4%)	44/47 (94%)	HAT1,IL1A,TINCR
Intraepithelial neoplasm	1.33	0.0638	0/47 (0%)	0/47 (0%)	3/47 (6%)	44/47 (94%)	ATF3,NBN,NKX3-1
Thoracic neoplasm	1.33	0.0638	0/47 (0%)	0/47 (0%)	3/47 (6%)	44/47 (94%)	CIITA,NMI,TNFSF10
Systemic inflammatory response syndrome and/or sepsis	1.33	0.0638	0/47 (0%)	0/47 (0%)	3/47 (6%)	44/47 (94%)	GBP5,ICAM1,STAT1
Endocrine pancreatic dysfunction	1.33	0.0638	1/47 (2%)	0/47 (0%)	2/47 (4%)	44/47 (94%)	CD274,SOCS1,ZBED6
Intrathoracic organ tumor	1.33	0.0638	0/47 (0%)	0/47 (0%)	3/47 (6%)	44/47 (94%)	CIITA,NMI,TNFSF10
Thrombocytosis	1.3	0.0625	0/48 (0%)	0/48 (0%)	3/48 (6%)	45/48 (94%)	IL6,JAK2,SLC2A3
Hematopoietic neoplasm	1.3	0.0625	0/48 (0%)	0/48 (0%)	3/48 (6%)	45/48 (94%)	CYP1B1,NMI,SLFN11
Sclerosis	1.3	0.0625	0/48 (0%)	0/48 (0%)	3/48 (6%)	45/48 (94%)	EDNRA,HSPE1,UGDH
Non-M3 acute myeloid leukemia	1.3	0.0625	0/48 (0%)	0/48 (0%)	3/48 (6%)	45/48 (94%)	BCL6,HSPE1,NMI
Endometrioid neoplasm	1.3	0.0625	0/48 (0%)	0/48 (0%)	3/48 (6%)	45/48 (94%)	CD274,FAS,HSPA6
Liquid tumor	1.3	0.0625	1/48 (2%)	0/48 (0%)	2/48 (4%)	45/48 (94%)	CD274,CEBPA,JAK2
Mediastinal lymphoma	1.3	0.0625	0/48 (0%)	0/48 (0%)	3/48 (6%)	45/48 (94%)	HSPE1,KLF4,NMI
Thorax lymphoma	1.3	0.0625	0/48 (0%)	0/48 (0%)	3/48 (6%)	45/48 (94%)	HSPE1,KLF4,NMI
Mature lymphocytic neoplasm	1.3	0.0625	0/48 (0%)	0/48 (0%)	3/48 (6%)	45/48 (94%)	BCL6,HSP90AA1,IL6
Acute coronary syndrome	1.3	0.0952	0/21 (0%)	0/21 (0%)	2/21 (10%)	19/21 (90%)	BACH1,TIA1
Genital tumor	1.28	0.0612	0/49 (0%)	0/49 (0%)	3/49 (6%)	46/49 (94%)	ATF3,MLF1,NBN
Pancreatic carcinoma	1.28	0.0612	1/49 (2%)	0/49 (0%)	2/49 (4%)	46/49 (94%)	SERPINB2,TOP1,VWF
Genital tract cancer	1.28	0.0612	1/49 (2%)	0/49 (0%)	2/49 (4%)	46/49 (94%)	ATF3,CEBPA,NBN
Pituitary lesion	1.28	0.0612	1/49 (2%)	0/49 (0%)	2/49 (4%)	46/49 (94%)	MLF1,SSTR5,TIA1
Large intestine carcinoma	1.28	0.0612	0/49 (0%)	0/49 (0%)	3/49 (6%)	46/49 (94%)	CD274,IL1A,PDCD1LG2
Hepatobiliary carcinoma	1.28	0.0612	0/49 (0%)	0/49 (0%)	3/49 (6%)	46/49 (94%)	IL1A,IL6,NMI
Pancreatobiliary adenocarcinoma	1.28	0.0612	0/49 (0%)	0/49 (0%)	3/49 (6%)	46/49 (94%)	CD274,IL6,TNFSF10
Alveolitis	1.26	0.0909	0/22 (0%)	0/22 (0%)	2/22 (9%)	20/22 (91%)	IL6,NAMPT
Edema	1.26	0.0909	1/22 (5%)	0/22 (0%)	1/22 (5%)	20/22 (91%)	CEBPA,SERPING1
Acidemia	1.26	0.0909	1/22 (5%)	0/22 (0%)	1/22 (5%)	20/22 (91%)	C4A/C4B,PRODH
Development of colorectal tumor	1.26	0.06	0/50 (0%)	0/50 (0%)	3/50 (6%)	47/50 (94%)	CD274,PDCD1LG2,SOCS1
Myeloid leukemia	1.26	0.06	1/50 (2%)	0/50 (0%)	2/50 (4%)	47/50 (94%)	CEBPA,EIF2S2,RIGI
Tachycardia	1.26	0.06	0/50 (0%)	0/50 (0%)	3/50 (6%)	47/50 (94%)	APOL6,HSPE1,SNRPE
Thymus cancer	1.26	0.06	1/50 (2%)	0/50 (0%)	2/50 (4%)	47/50 (94%)	HSPE1,NMI,SSTR5
Bone marrow cancer	1.26	0.06	1/50 (2%)	0/50 (0%)	2/50 (4%)	47/50 (94%)	CEBPA,EIF2S2,RIGI
Upper gastrointestinal tract cancer	1.26	0.06	1/50 (2%)	0/50 (0%)	2/50 (4%)	47/50 (94%)	ME1,NBN,PRODH
Bone marrow neoplasm	1.26	0.06	1/50 (2%)	0/50 (0%)	2/50 (4%)	47/50 (94%)	CEBPA,JAK2,RIGI
Malignant neoplasm of anterior mediastinum	1.26	0.06	1/50 (2%)	0/50 (0%)	2/50 (4%)	47/50 (94%)	HSPE1,NMI,SSTR5
Malignant neoplasm of heart, mediastinum and pleura	1.26	0.06	0/50 (0%)	0/50 (0%)	3/50 (6%)	47/50 (94%)	HSPE1,KLF4,NMI
Connective tissue cancer	1.26	0.06	0/50 (0%)	0/50 (0%)	3/50 (6%)	47/50 (94%)	MLF1,NMI,TOP1
Mediastinal cancer	1.26	0.06	0/50 (0%)	0/50 (0%)	3/50 (6%)	47/50 (94%)	HSPE1,KLF4,NMI
Aplastic anemia	1.24	0.0588	1/51 (2%)	0/51 (0%)	2/51 (4%)	48/51 (94%)	CD74,NOXO1,TXNRD1
Lung adenocarcinoma	1.24	0.0588	1/51 (2%)	0/51 (0%)	2/51 (4%)	48/51 (94%)	CEBPA,MLF1,NMI
Ear malformation	1.24	0.0588	1/51 (2%)	0/51 (0%)	2/51 (4%)	48/51 (94%)	IRF1,PRRX2,SP110
B-cell lymphoma	1.24	0.0588	0/51 (0%)	0/51 (0%)	3/51 (6%)	48/51 (94%)	BCL6,CUL2,NBN
Autosomal dominant polycystic kidney disease	1.23	0.087	1/23 (4%)	0/23 (0%)	1/23 (4%)	21/23 (91%)	ATF3,SSTR5
Nonalcoholic steatohepatitis	1.23	0.087	1/23 (4%)	0/23 (0%)	1/23 (4%)	21/23 (91%)	BARX2,EIF2S2
Microangiopathy	1.23	0.087	1/23 (4%)	0/23 (0%)	1/23 (4%)	21/23 (91%)	CFH,VWF
Testicular tumor	1.22	0.0577	0/52 (0%)	0/52 (0%)	3/52 (6%)	49/52 (94%)	CUL2,HSP90AA1,LYN
Angiosarcoma	1.22	0.0577	0/52 (0%)	0/52 (0%)	3/52 (6%)	49/52 (94%)	IL6,IRF1,NMI
Malignant intracranial tumor	1.2	0.0566	0/53 (0%)	0/53 (0%)	3/53 (6%)	50/53 (94%)	FERMT2,RAB23,SLFN11
Development of genital tumor	1.2	0.0566	1/53 (2%)	0/53 (0%)	2/53 (4%)	50/53 (94%)	CEBPA,KLF4,NBN

Retinitis	1.2	0.0566	0/53 (0%)	0/53 (0%)	3/53 (6%)	50/53 (94%)	CD274,STAT1,TNFRSF1B
Scar tissue	1.2	0.0566	1/53 (2%)	0/53 (0%)	2/53 (4%)	50/53 (94%)	CTNNAL1,CXCL10,KRT15
Nonsquamous non-small cell lung carcinoma	1.2	0.0566	1/53 (2%)	0/53 (0%)	2/53 (4%)	50/53 (94%)	CEBPA,MLF1,NMI
Malignant neoplasm of eye	1.2	0.0566	0/53 (0%)	0/53 (0%)	3/53 (6%)	50/53 (94%)	HSP90AA1,SNRPE,TOP1
Malignant neoplasm of eye and adnexa	1.2	0.0566	0/53 (0%)	0/53 (0%)	3/53 (6%)	50/53 (94%)	HSP90AA1,SNRPE,TOP1
Extrapancreatic neuroendocrine tumor	1.2	0.0566	0/53 (0%)	0/53 (0%)	3/53 (6%)	50/53 (94%)	CD274,MLF1,TIA1
Non-squamous lung carcinoma	1.2	0.0566	1/53 (2%)	0/53 (0%)	2/53 (4%)	50/53 (94%)	CEBPA,MLF1,NMI
Orbital cancer	1.2	0.0566	0/53 (0%)	0/53 (0%)	3/53 (6%)	50/53 (94%)	HSP90AA1,SNRPE,TOP1
Non-squamous lung cancer	1.2	0.0566	1/53 (2%)	0/53 (0%)	2/53 (4%)	50/53 (94%)	CEBPA,MLF1,NMI
Arrhythmia	1.2	0.0833	0/24 (0%)	0/24 (0%)	2/24 (8%)	22/24 (92%)	RGS7,SNRPE
Diffuse B-cell lymphoma	1.18	0.0556	0/54 (0%)	0/54 (0%)	3/54 (6%)	51/54 (94%)	BCL6,KLF4,NOC3L
Pituitary gland adenoma	1.18	0.0556	1/54 (2%)	0/54 (0%)	2/54 (4%)	51/54 (94%)	DNAJA4,FERMT2,SSTR5
Polycystic Kidney Disease	1.18	0.0556	2/54 (4%)	0/54 (0%)	1/54 (2%)	51/54 (94%)	ATF3,CKB,SSTR5
Sellar neoplasm	1.18	0.0556	1/54 (2%)	0/54 (0%)	2/54 (4%)	51/54 (94%)	DNAJA4,FERMT2,SSTR5
Familial congenital anomalies of the kidney and urinary tract	1.18	0.0556	2/54 (4%)	0/54 (0%)	1/54 (2%)	51/54 (94%)	ATF3,CKB,SSTR5
Hypersensitivity reaction type I	1.16	0.08	1/25 (4%)	0/25 (0%)	1/25 (4%)	23/25 (92%)	NOXO1,ZFP36
Infarction of heart	1.16	0.08	1/25 (4%)	0/25 (0%)	1/25 (4%)	23/25 (92%)	TNFRSF1B,VWF
Infarction of cardiac muscle	1.16	0.08	1/25 (4%)	0/25 (0%)	1/25 (4%)	23/25 (92%)	TNFRSF1B,VWF
Myocardial infarction	1.16	0.08	1/25 (4%)	0/25 (0%)	1/25 (4%)	23/25 (92%)	TNFRSF1B,VWF
Autosomal dominant kidney disease	1.16	0.08	1/25 (4%)	0/25 (0%)	1/25 (4%)	23/25 (92%)	ATF3,SSTR5
Brainstem disorder	1.16	0.08	0/25 (0%)	0/25 (0%)	2/25 (8%)	23/25 (92%)	CDKN3,HSPA1A/HSPA1B
Endocrine gland tumor	1.16	0.0545	0/55 (0%)	0/55 (0%)	3/55 (5%)	52/55 (95%)	IRF1,MLF1,TIA1
Dysfunction of left ventricle	1.16	0.0545	1/55 (2%)	0/55 (0%)	2/55 (4%)	52/55 (95%)	CSF1,SREBF1,UGDH
B-cell non-Hodgkin lymphoma	1.16	0.0545	0/55 (0%)	0/55 (0%)	3/55 (5%)	52/55 (95%)	BCL6,HSP90AA1,NOC3L
Lung carcinoma	1.16	0.0545	0/55 (0%)	0/55 (0%)	3/55 (5%)	52/55 (95%)	MLF1,NMI,TNFSF10
Myeloproliferative neoplasm	1.14	0.0536	1/56 (2%)	0/56 (0%)	2/56 (4%)	53/56 (95%)	JAK2,NOTCH3,RIGI
Thrombus	1.13	0.0769	1/26 (4%)	0/26 (0%)	1/26 (4%)	24/26 (92%)	CFH,VWF
Lung injury	1.13	0.0769	0/26 (0%)	0/26 (0%)	2/26 (8%)	24/26 (92%)	IL6,JAK2
Depigmentation/hyperpigmentation of skin	1.13	0.0769	0/26 (0%)	0/26 (0%)	2/26 (8%)	24/26 (92%)	GBP5,NBN
Myosarcoma	1.12	0.0526	0/57 (0%)	0/57 (0%)	3/57 (5%)	54/57 (95%)	HMOX1,NMI,WARS1
Aggressive cancer	1.12	0.0526	0/57 (0%)	0/57 (0%)	3/57 (5%)	54/57 (95%)	BCL6,KLF4,NOC3L
Primary myelofibrosis	1.12	0.0526	0/57 (0%)	0/57 (0%)	3/57 (5%)	54/57 (95%)	HSP90AA1,JAK2,UGDH
Development of lung carcinoma	1.11	0.0517	1/58 (2%)	0/58 (0%)	2/58 (3%)	55/58 (95%)	CEBPA,MLF1,NMI
Skin carcinoma	1.11	0.0517	0/58 (0%)	0/58 (0%)	3/58 (5%)	55/58 (95%)	CD274,FERMT2,MLF1
Myeloid neoplasm	1.11	0.0517	0/58 (0%)	0/58 (0%)	3/58 (5%)	55/58 (95%)	EIF2S2,JAK2,RIGI
Remodeling of left ventricle	1.11	0.0741	0/27 (0%)	0/27 (0%)	2/27 (7%)	25/27 (93%)	ANXA1,FAS
Acute coronary event	1.11	0.0741	0/27 (0%)	0/27 (0%)	2/27 (7%)	25/27 (93%)	BACH1,TIA1
Rhizomelia	1.11	0.0741	2/27 (7%)	0/27 (0%)	0/27 (0%)	25/27 (93%)	FGF1,MMP13
Ventricular remodeling	1.11	0.0741	0/27 (0%)	0/27 (0%)	2/27 (7%)	25/27 (93%)	ANXA1,FAS
Bone resorption disorder	1.11	0.0741	0/27 (0%)	0/27 (0%)	2/27 (7%)	25/27 (93%)	EIF2S2,IL1A
Development of head and neck tumor	1.09	0.0508	0/59 (0%)	0/59 (0%)	3/59 (5%)	56/59 (95%)	EIF2S2,HSP90AA1,TOP1
Lymphomagenesis of T lymphocytes	1.09	0.0508	0/59 (0%)	0/59 (0%)	3/59 (5%)	56/59 (95%)	NBN,NMI,SLFN11
T-cell non-Hodgkin lymphoma	1.09	0.0508	0/59 (0%)	0/59 (0%)	3/59 (5%)	56/59 (95%)	NBN,NMI,SLFN11
Osteosarcoma	1.09	0.0508	0/59 (0%)	0/59 (0%)	3/59 (5%)	56/59 (95%)	MLF1,NMI,TOP1
Development of malignant neoplasm of lung	1.09	0.0508	1/59 (2%)	0/59 (0%)	2/59 (3%)	56/59 (95%)	CEBPA,MLF1,NMI
Autosomal recessive inborn error of metabolism	1.08	0.0714	0/28 (0%)	0/28 (0%)	2/28 (7%)	26/28 (93%)	CIITA,NBN
Cerebral edema	1.08	0.0714	0/28 (0%)	0/28 (0%)	2/28 (7%)	26/28 (93%)	HMOX1,HSPE1
Idiopathic and/or familial pulmonary arterial hypertension	1.08	0.0714	0/28 (0%)	0/28 (0%)	2/28 (7%)	26/28 (93%)	EDNRA,HIF1A
High-grade lymphoma	1.07	0.05	0/60 (0%)	0/60 (0%)	3/60 (5%)	57/60 (95%)	BCL6,KLF4,NOC3L
Epileptic seizure	1.07	0.05	0/60 (0%)	0/60 (0%)	3/60 (5%)	57/60 (95%)	GEMIN2,SMN1/SMN2,UGDH
Development of non-small cell lung carcinoma	1.07	0.05	1/60 (2%)	0/60 (0%)	2/60 (3%)	57/60 (95%)	CEBPA,MLF1,NMI
Uterine tumor	1.06	0.0492	1/61 (2%)	0/61 (0%)	2/61 (3%)	58/61 (95%)	CEBPA,NBN,TAP1
Non-small cell adenocarcinoma	1.06	0.0492	1/61 (2%)	0/61 (0%)	2/61 (3%)	58/61 (95%)	CEBPA,MLF1,NMI
Head and neck neuroendocrine tumor	1.06	0.0492	1/61 (2%)	0/61 (0%)	2/61 (3%)	58/61 (95%)	MLF1,SSTR5,TIA1
Thyroiditis	1.05	0.069	0/29 (0%)	0/29 (0%)	2/29 (7%)	27/29 (93%)	ANXA1,FAS
Infarction	1.05	0.069	0/29 (0%)	0/29 (0%)	2/29 (7%)	27/29 (93%)	HMOX1,TNFRSF1B
Primary pulmonary hypertension	1.05	0.069	0/29 (0%)	0/29 (0%)	2/29 (7%)	27/29 (93%)	EDNRA,HIF1A
Diastolic dysfunction	1.05	0.069	0/29 (0%)	0/29 (0%)	2/29 (7%)	27/29 (93%)	IL1A,IL6
Visceromegaly	1.05	0.069	0/29 (0%)	0/29 (0%)	2/29 (7%)	27/29 (93%)	CUL2,MLF1
Corneal neovascularization	1.05	0.069	0/29 (0%)	0/29 (0%)	2/29 (7%)	27/29 (93%)	ICAM1,IL1A
Pituitary gland tumor	1.04	0.0484	1/62 (2%)	0/62 (0%)	2/62 (3%)	59/62 (95%)	MLF1,SSTR5,TIA1

Immune mediated inflammatory disease	1.03	0.0667	0/30 (0%)	0/30 (0%)	2/30 (7%)	28/30 (93%)	BCL6,TRIM21
Chronic renal failure	1.03	0.0667	1/30 (3%)	0/30 (0%)	1/30 (3%)	28/30 (93%)	CCDC88B,TNFRSF1B
Hypoalbuminemia	1.03	0.0667	0/30 (0%)	0/30 (0%)	2/30 (7%)	28/30 (93%)	B2M,PMAIP1
Spinal muscular atrophy	1.03	0.0667	0/30 (0%)	0/30 (0%)	2/30 (7%)	28/30 (93%)	GEMIN2,SMN1/SMN2
Peripheral vascular disease	1.03	0.0667	2/30 (7%)	0/30 (0%)	0/30 (0%)	28/30 (93%)	NOTCH3,VWF
Lower motor neuron disease	1.03	0.0667	0/30 (0%)	0/30 (0%)	2/30 (7%)	28/30 (93%)	GEMIN2,SMN1/SMN2
Immunoglobulin deficiency	1.03	0.0667	0/30 (0%)	0/30 (0%)	2/30 (7%)	28/30 (93%)	C4A/C4B,LYN
Axial myopathy	1.03	0.0667	0/30 (0%)	0/30 (0%)	2/30 (7%)	28/30 (93%)	GEMIN2,SMN1/SMN2
Severe encephalopathy	1.03	0.0667	0/30 (0%)	0/30 (0%)	2/30 (7%)	28/30 (93%)	C4A/C4B,DNAJA4
Antibody-deficiency syndrome	1.03	0.0667	0/30 (0%)	0/30 (0%)	2/30 (7%)	28/30 (93%)	C4A/C4B,LYN
Growth failure or short stature	1.03	0.0667	1/30 (3%)	0/30 (0%)	1/30 (3%)	28/30 (93%)	CEBPA,NOCT
Chronic renal impairment	1.03	0.0667	1/30 (3%)	0/30 (0%)	1/30 (3%)	28/30 (93%)	CCDC88B,TNFRSF1B
Progression of squamous-cell carcinoma	1.01	0.0469	1/64 (2%)	0/64 (0%)	2/64 (3%)	61/64 (95%)	DUSP1,SP100,SSTR5
Progression of squamous cell tumor	1.01	0.0469	1/64 (2%)	0/64 (0%)	2/64 (3%)	61/64 (95%)	DUSP1,SP100,SSTR5
Periodontal disease	1	0.0645	0/31 (0%)	0/31 (0%)	2/31 (6%)	29/31 (94%)	C1R,IL6
End stage renal disease	1	0.0645	1/31 (3%)	0/31 (0%)	1/31 (3%)	29/31 (94%)	CCDC88B,TNFRSF1B
Chronic pulmonary disease	1	0.0645	0/31 (0%)	0/31 (0%)	2/31 (6%)	29/31 (94%)	CTSS,STAT1
Epithelial ovarian tumor	1	0.0645	0/31 (0%)	0/31 (0%)	2/31 (6%)	29/31 (94%)	B2M,IRF1
Chronic skin disorder	1	0.0645	0/31 (0%)	0/31 (0%)	2/31 (6%)	29/31 (94%)	IL1A,STAT1
Extracranial solid tumor	1	0.0645	0/31 (0%)	0/31 (0%)	2/31 (6%)	29/31 (94%)	NMI,TNFSF10
Advanced-stage chronic kidney disease	1	0.0645	1/31 (3%)	0/31 (0%)	1/31 (3%)	29/31 (94%)	CCDC88B,TNFRSF1B
Ovarian or fallopian tube carcinoma	1	0.0645	0/31 (0%)	0/31 (0%)	2/31 (6%)	29/31 (94%)	B2M,IRF1
